# Relationships between Inhibition, Transport and Enhanced Transport via the Organic Cation Transporter 1

**DOI:** 10.3390/ijms23042007

**Published:** 2022-02-11

**Authors:** Ole Jensen, Lukas Gebauer, Jürgen Brockmöller, Christof Dücker

**Affiliations:** Institute of Clinical Pharmacology, University Medical Center Göttingen, D-37075 Göttingen, Germany; ole.jensen@med.uni-goettingen.de (O.J.); lukas.gebauer@med.uni-goettingen.de (L.G.); jbrockm@gwdg.de (J.B.)

**Keywords:** OCT1, inhibition, transport, potentiation, enhancement, *SLC22A1*

## Abstract

The organic cation transporter 1 (OCT1, *SLC22A1*) transports a large number of structurally diverse endogenous and exogenous substrates. There are numerous known competitive and non-competitive inhibitors of OCT1, but there are no studies systematically analyzing the relationship between transport, stimulation, and inhibition. Here, we tested in vitro OCT1 inhibition by OCT1 substrates and transport of OCT1 inhibitors under uniform analytical conditions. Beyond inhibition testing with two model substrates, we tested nine additional OCT1 substrates for their mutual inhibition. Inhibition of ASP^+^ uptake by most OCT1 substrates was weak. The model substrate sumatriptan, with its moderately stronger inhibitability, was used to confirm this. Interestingly, OCT1 substrates exhibiting stronger OCT1 inhibition were mainly biaromatic β-agonistic drugs, such as dobutamine, fenoterol, ractopamine and ritodrine. Biaromatic organic cations were both, strong inhibitors and good substrates, but many OCT1 substrates showed little pairwise inhibition. Surprisingly, sumatriptan did significantly enhance dobutamine uptake. This effect was concentration dependent and additional experiments indicated that efflux inhibition may be one of the underlying mechanisms. Our data suggests, that OCT1 substrates are mainly weak OCT1 inhibitors and among those inhibiting well, noncompetitive inhibition could be responsible. Weak competitive inhibition confirms that OCT1 inhibition screenings poorly predict OCT1 substrates. Additionally, we showed that the OCT1 substrate sumatriptan can enhance uptake of some other OCT1 substrates. OCT1 transport stimulation was already observed earlier but is still poorly understood. Low OCT1 uptake inhibition and strong OCT1 efflux inhibition could be mechanisms exploitable for enhancing transport.

## 1. Introduction

OCT1 is the most strongly expressed organic cation transporter in the sinusoidal membrane of the human liver [1,2]. Many endogenous and exogenous substances, among them many drugs, are either inhibitors or substrates of OCT1, or both [3,4,5,6,7,8,9,10,11]. OCT1 is genetically highly polymorphic and frequent variants have reduced transport activity or complete loss of function. With some drug–substrates of OCT1, deficient or very low activity of this transporter may have contributed to the access mortality of the respective drug [12]. Modulation of pharmacokinetics, drug effects or adverse drug reactions by OCT1 polymorphisms have been shown for a number of drugs [5,7,8,9]. In addition, substances that inhibit OCT1 could mimic genetic deficiency (so-called phenocopies). While the role of OCTs in drug–drug interactions is understood quite well, there is limited knowledge about the association of OCTs with diseases, with only OCTN2 deficiency being linked to a syndrome [3,13]. The physiological role of most OCTs remains uncertain, at least, complete loss of OCT1 activity is not associated with major pathologies. In part, loss of OCT activity may be compensated by other polyspecific transporters.

Known OCT1 substrates are highly variable in their molecular structures [3]. The physicochemical and structural features of OCT1 substrates have been studied extensively [3,10,14,15,16]. Typical substrates of OCT1 are on average smaller than 500 Å^3^ in volume [10], have a positively charged nitrogen, an aromatic ring, and moieties which increase hydrophilicity [3]. In contrast, inhibitors of OCT1 are less often positively charged and more lipophilic [11]. Thus, there is both an overlap and a symmetric difference between OCT1 substrates and inhibitors, which is currently not entirely understood. There are well-known OCT1 inhibitors that are also substrates of OCT1. However, other well-known OCT1 inhibitors may either not be substrates or cannot be proven to be substrates because regular uptake assays are insensitive towards highly lipophilic compounds, where net uptake is masked by high passive diffusion. Irrespective of these unanswered questions, numerous potent inhibitors have been excluded as substrates, proving that substrates and inhibitors are intertwined, but not necessarily the same [3].

There is ample data on OCT1 inhibition, but it is mainly based on inhibition of a few OCT1 model substrates. On the other hand, multiple substrate binding sites and non-competitive OCT1 inhibition have been identified [3,17,18,19]. For instance, data on rat Oct1 for 1-methyl-4-phenylpyridinium (MPP^+^) suggests the existence of two low-affinity binding sites and one high-affinity binding site with allosteric inhibitory effects on the low-affinity binding sites [20]. Such specific inhibitory binding sites would discourage from screening for OCT1 transport by using inhibition assays with a few model substrates but, to our knowledge, the relationship between substrate and inhibitory properties of OCT1 substrates has not yet been investigated based on large datasets integrating inhibition as well as transport data. Thus, while there is good knowledge on OCT1 inhibitors and substrates, much less is known about the OCT1 inhibition by OCT1 substrates and about differences between substrate and non-substrate inhibitors of OCT1. Comprehensive knowledge of this will be indispensable when it comes to predicting drug–drug interactions. Bioinformatic prediction is impeded by the lack of an OCT1 crystal structure, reducing structure-based approaches to homology modelling [21,22]. Currently, a better understanding of OCT1 function may be achieved with less uncertainty by ligand-based experimental and bioinformatics approaches.

In this study, we tested in vitro OCT1 substrates for inhibition and, in particular, we tested in vitro a large number of OCT1 inhibitors for their transport via OCT1. We compared inhibition results for two substrates of OCT1, the standard model substrate ASP^+^ and the clinically relevant substrate sumatriptan. For a subset of OCT1 substrates, we compared their inhibition and inhibitability, with the latter meaning how strongly the distribution and/or elimination can be inhibited, as studied based on pairs of substances. Based on those results, we picked candidate pairs to discover a possible cotransport and improvement of one compound’s transport by presence of a second compound (enhanced transport, also known as potentiation, augmentation or stimulation) and indeed, we found significant enhancement of OCT1-mediated transport with some specific pairs of substrates. Finally, we performed experiments, the results of which suggest that inhibition of OCT1-mediated efflux by one compound could be the mechanism leading to increased OCT-1 mediated uptake of the second compound.

## 2. Results

**Figure 1:**
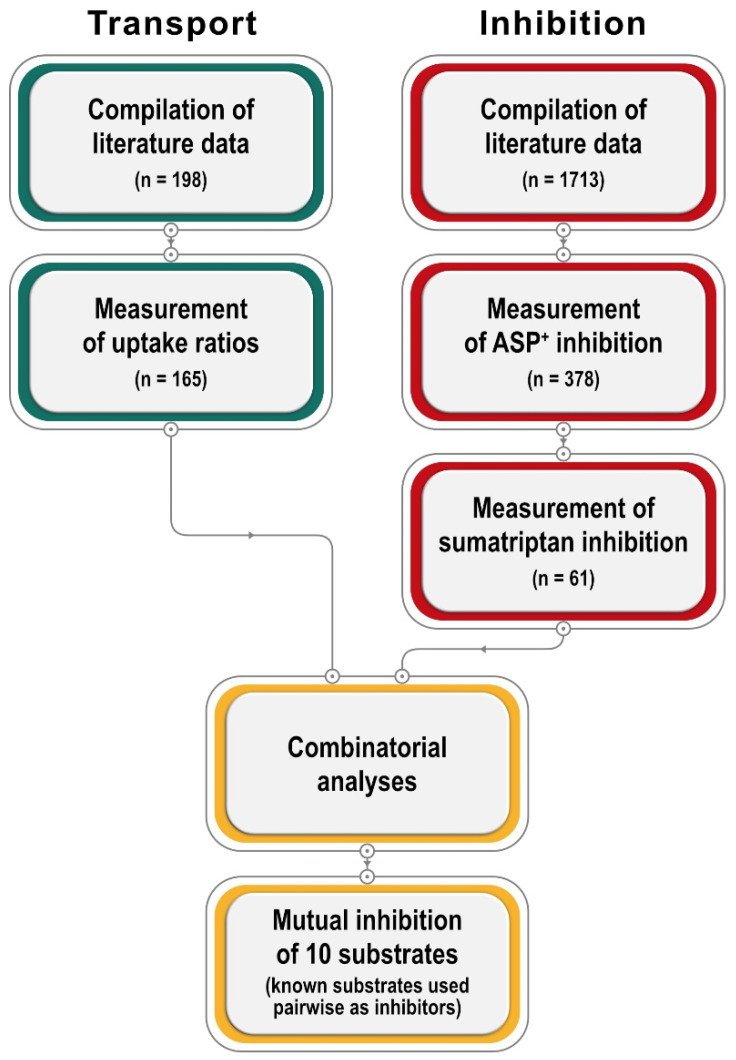
Chart summarizing the general workflow of this publication. Literature data was used for inter-laboratory cross-validation.

We present a large database on inhibition and transport by OCT1, and subsequent experiments explaining their relationship (Figure 1). First, we extended the OCT1 substrate database using a unified parameter for measuring transport, the ratio of uptake in overexpressing cells over uptake in mock-transfected cells. Second, we extended the list of OCT1 inhibitors using the standard model substrate ASP^+^ and, in addition, sumatriptan, one of the most specific and clinically relevant OCT1 substrates. We added new in vitro data where no in vitro data with comparable methodology was available. Third, based on the inhibition and transport data, we analyzed the relationship between both. In addition, we analyzed mutual inhibition between 10 characteristic substrates of OCT1. Excitingly, these analyses revealed compound pairs with enhanced transport as well as pairs showing no inhibition, so that, fourth, we analyzed those pairs in more detail using a wide range of concentrations. As underlined by our final experiments, uptake enhancement may be explained by inhibition of substrate efflux after successful uptake.

### 2.1. OCT1 Substrates

**Figure 2:**
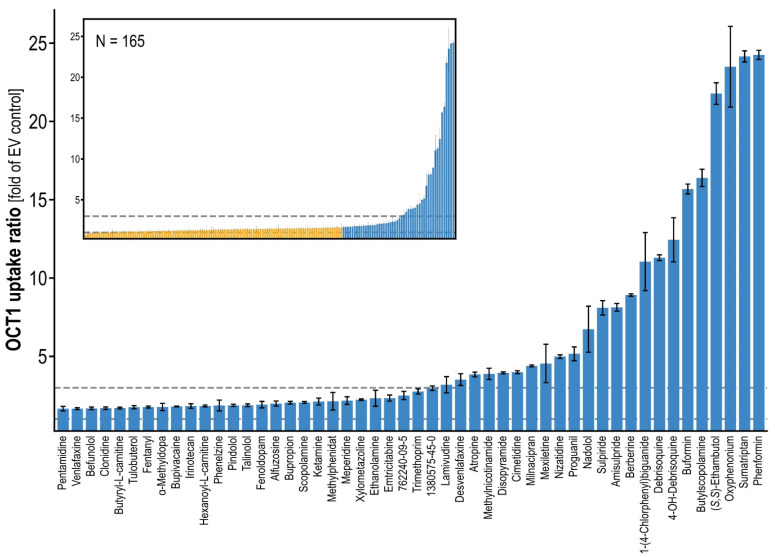
Substrate uptake ratios in OCT1 overexpressing cells over empty vector cells at 2.5 µM. Error bars indicate standard error of the mean between replicates. The large plot shows the top 50 substrates, the miniature plot shows the overview of all 165 tested compounds. The upper dashed line indicates the uptake ratio of 3, considered by the authors as a reasonable cutoff for relevant transport.

For known OCT1 substrates, for which no uptake ratios were available to us, we performed in vitro transport assays at a concentration of 2.5 µM, as established and applied previously [10,23]. This concentration was considered a trade-off between linearity in sub-*K*_m_ concentrations and detectability in mass spectrometric analysis, and it had been used in larger comparable screenings before [10]. In total, we analyzed OCT1 transport of 165 compounds, which were selected first based on typical characteristics of OCT1 substrates, and, second, to cover a wide range of structural diversity (Figure 2) [3]. This included previously known OCT1 substrates now tested with our methodology. This resulted in a total of 326 compounds (including published data), for which uptake ratios were determined in a uniform manner (Figure S2). When taking a conservative ratio of 3 as a cutoff for the differentiation between substrate and non-substrate as applied in previous publications [16,23], the overall database now included 84 substrates (most already known as substrates of OCT1). When using the less conservative criterion of a twofold increase by transporter-mediated uptake, which is suggested by the FDA, that number was 107. Within these, interesting novel OCT1 substrates were found, such as CAS 762240-09-5 and CAS 1380575-45-0, which are fragments of the tyrosine kinase inhibitors crizotinib and ceritinib (cf. Table S1), revealing uptake ratios of 2.3 and 2.7, respectively. In addition, the antiarrhythmic agent mexiletine showed an uptake ratio of 4.3. Interestingly and in line with a prior publication, the anionic HIV reverse-transcriptase inhibitor lamivudine revealed an uptake ratio of 3.0 [24]. The top OCT1 substrates tested in this study (newly found as well as previously known) are listed in Table S2.

### 2.2. OCT1 Inhibition

**Figure 3:**
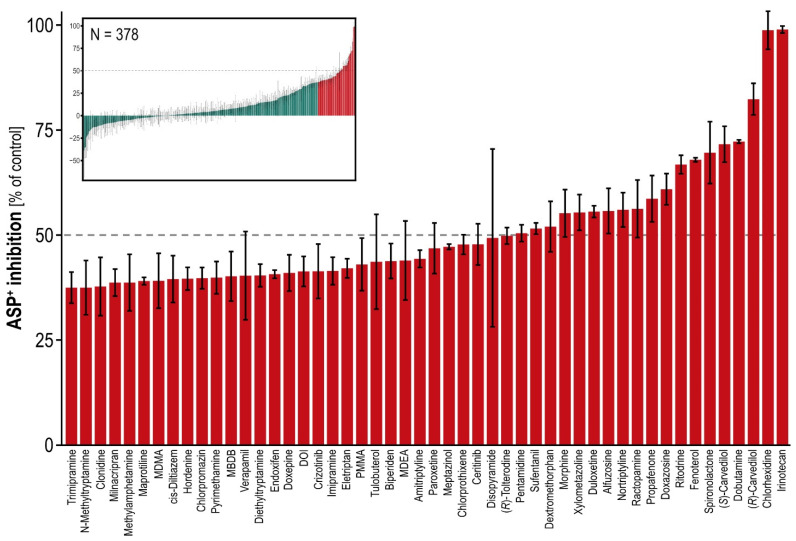
Percentage inhibition of OCT1-mediated ASP^+^ transport by various compounds. Authors’ own, unpublished data. Error bars indicate standard error of the mean between replicates. The large plot shows the top 50 inhibitors, the miniature plot shows the overview of all 378 tested compounds.

All available known OCT1 substrates and additional structurally diverse compounds were screened as inhibitors of the model substrate ASP^+^. The screening library included both already published and newly identified OCT1 substrates, and published as well as newly identified OCT1 inhibitors. Maximum inhibition was found with irinotecan, which inhibited OCT1 activity by 99%, whereas the broad inhibitor of numerous transporters and cytochrome P450 enzymes, verapamil, inhibited OCT1 only by about 50% (Figure 3, Table S3).

Among the “negatively inhibiting” (in other words transport enhancing) compounds, bisoprolol (−38.8%), flunarizine (−35.0%) and famotidine (−20.4%) had relatively low standard errors and seemed indeed to be enhancers of transport. This finding was, however, not highly reproducible among laboratories (Table S2). Bisoprolol, for example, had only a slightly negative inhibition in another screening (−3.36) [17].

In general, our results match the picture from earlier inhibition screenings, where inhibition ranged from −26% to around 100%, despite slight disparities in methodologies (Figure S2). Irrespective of the unanswered questions concerning enhancement (“negative inhibition”), data from several laboratories indicated that such an enhancement may be a real mechanism with potential impact on transport kinetics of drugs and of endogenous substances.

**Figure 4:**
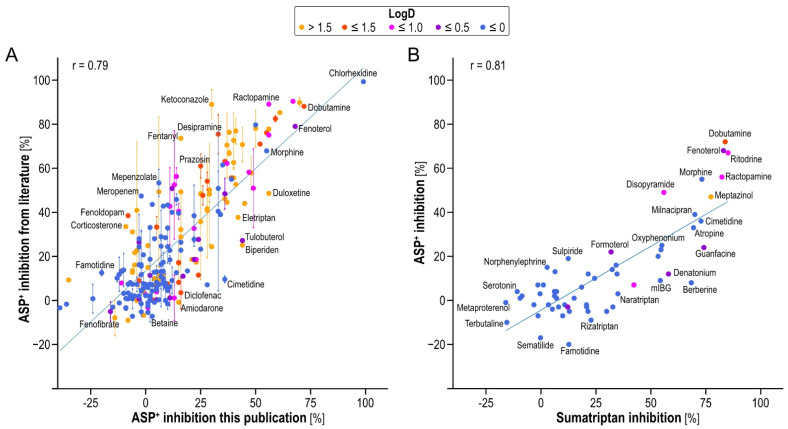
(**A**) Comparison of OCT1 inhibition as measured by reduction in ASP^+^ uptake in the present study with measurements published earlier [11,17]. (**B**) Comparison of OCT1 inhibition as measured by reduction in sumatriptan uptake with the respective inhibition measured with ASP+. Dots indicate the means, vertical lines the range between laboratories. Dot color is indicating logD of the respective compounds at pH 7.4. Only selected substances are labelled, a complete listing of all our data is given in Table S1.

We correlated our inhibition data with the available literature data to analyze the reproducibility [11,17] (Figure 4A). As seen, and as known in the field, inter-laboratory reproducibility is not perfect but no systematic deviation from the line of unity was observed. The slope of the regression line was 0.93, sufficiently close to 1, and had a correlation coefficient of 0.79.

Since inhibition may be substrate-dependent, we also studied inhibition using sumatriptan as a second OCT1 model substrate. While sumatriptan was, in general, inhibited more strongly compared to ASP^+^ (slope of the regression line only 0.58), the correlation was nonetheless linear and numerically even higher than the inter-laboratory reproducibility with the same substrate (r = 0.81, Figure 4B).

In general, OCT1 inhibition by OCT1 substrates was weak, but with some notable exceptions consistent across both model substrates. Interestingly, the structurally similar biaromatic β-agonists ritodrine, dobutamine, fenoterol and ractopamine showed the highest inhibition among all inhibitors tested here with both substrates, ASP^+^ and sumatriptan. Among non-substrates, rather lipophilic substances such as spironolactone and doxazosin were confirmed as strong inhibitors. Among the hydrophilic non-substrates, the antiseptic chlorhexidine was confirmed as the strongest OCT1 inhibitor.

### 2.3. Relation between Transport and Inhibition

**Figure 5:**
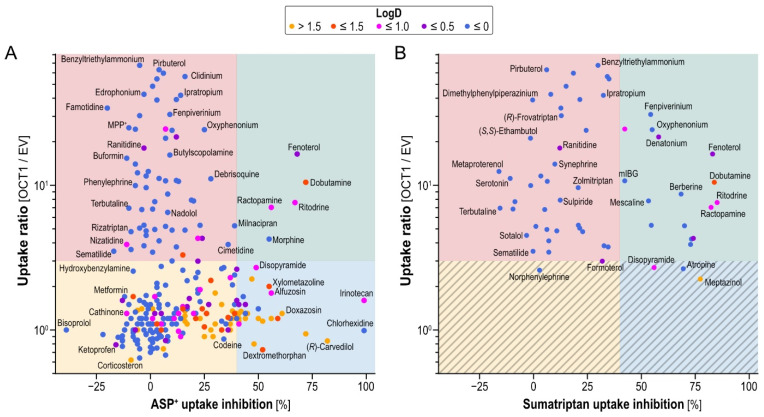
OCT1 uptake ratio (means of new and previously published data) versus inhibition data. (**A**) Inhibition data using model substrate ASP^+^. (**B**) Inhibition data using model substrate sumatriptan. Dot color indicates logD of the respective compounds at pH 7.4. Colored quadrants indicate non-inhibiting substrates (red), inhibiting substrates (green), inhibiting non-substrates (blue) and non-inhibiting non-substrates (yellow). Most substrates with an uptake ratio < 3 have not been tested with sumatriptan as indicated by the hatched areas in (**B**).

Our study aimed to elucidate the relationship between structures transported by OCT1 and structures inhibiting OCT1. As illustrated in Figure 5A (ASP^+^) and Figure 5B (sumatriptan), we found all combinations of OCT1 transport, non-transport, inhibition and non-inhibition.

The majority of tested compounds showed neither OCT1-mediated uptake, ratio ≤ 3, nor OCT1-inhibition, >40%. We decided on a cutoff of 40% inhibition instead of 50%, as often applied, because of the reduced inhibitor concentration used, compared with other OCT1 inhibition screenings [11,17]. These compounds can therefore be categorized as non-substrate-non-inhibitors (yellow quadrant in Figure 5A). Surprisingly, among the tested OCT1 substrates, the majority did not inhibit either model substrate with the concentrations used here (2 µM for substrates and 20 µM for potential inhibitors; red quadrants in Figure 5), making them substrate-non-inhibitors. For example, drugs such as pirbuterol, terbutaline and sematilide showed no relevant OCT1 inhibition for either of the model substrates, at least not at the concentrations used in our assays. Fenoterol, dobutamine, ritodrine and ractopamine showed the strongest inhibition (≥40%) of OCT1-mediated ASP^+^ as well as sumatriptan transport (upper-right quadrants of Figure 5A,B), making them substrate-inhibitors of OCT1 (green quadrants in Figure 5). As tested with ASP^+^ as model substrate only, there were many non-OCT1-substrates (uptake ratios < 3), that strongly inhibited OCT1-mediated uptake (blue quadrant in Figure 5A).

Comparing ASP^+^ and sumatriptan inhibition, as can be seen from the greater abundance of compounds in the upper right quadrant of Figure 5B than in the right upper quadrant of Figure 5A, sumatriptan uptake could in general be better inhibited by the OCT1 substrates tested.

Chemical characterization of non-substrate-non-inhibitors, substrate-non-inhibitors, substrate-inhibitors and non-substrate-inhibitors of OCT1-mediated ASP^+^ uptake is given in Table 1.

In short, the molecular weight was significantly different between groups (*p* < 0.01, Kruskal–Wallis test), and inhibitors were found to be heavier than non-inhibitors regardless of transport rate. The logD value at pH 7.4 differed significantly (*p* < 0.01) and inhibitors were more lipophilic than non-inhibitors among all substrates of OCT1. Non-substrate-non-inhibitors were less positively charged compared to other groups of substrates and inhibitors. Ring count differed significantly between groups (*p* < 0.01) and was higher in inhibitors than in non-inhibitors. In contrast, the number H bond donors, which was significantly different between groups as well (*p* < 0.01), was higher in good substrates than in poor or non-substrates. A similar analysis was performed for sumatriptan inhibition and can be found in Table S4.

With the concentration of the model substrate far below its *K*_m_, assuming competitive inhibition, the Cheng–Prusoff equation predicts the IC_50_ to be approximately identical to the *K*_i_. Many of the inhibitor candidates tested had previously been reported with apparent *K*_m_ values smaller than the concentration used in our assay (20 µM). For substrate-inhibitors *K*_m_ and *K*_i_ are supposed to be equal in case the mode of inhibition relies solely on competitive inhibition at the same binding site [25]. For the inhibitor candidates with *K*_m_ values smaller than the concentration used, this suggests an inhibition of at least 50%. However, for a selection of inhibitor candidates, experimentally found inhibition was strikingly lower in nearly 50% of the substances tested (Table 2). To further elucidate the complex interaction of OCT1 substrates and inhibitors, we tested a selection of ten substrate-inhibitors and substrate-non-inhibitors for mutual inhibition of the other nine compounds.

### 2.4. Mutual Substrate Inhibition

**Figure 6:**
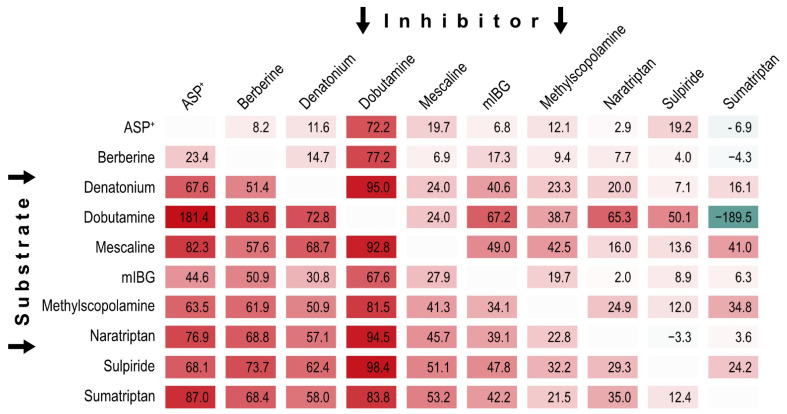
Mutual inhibition of ten selected OCT1-substrates. Data provided as percentage inhibition compared to uptake into non-inhibited OCT1 overexpressing and EV-transfected cells. Cell colors corresponding to degree of inhibition. It may be noted that negative inhibition corresponds to stimulation of transport (sumatriptan effect on dobutamine) while inhibition of more than 100% (ASP^+^ effect on dobutamine) might correspond to ASP^+^-mediated stimulation of dobutamine efflux.

Considering ample evidence for multiple binding sites of OCT1 substrates and inhibitors, we performed pairwise inhibition testing, also with the intention to find mutually non-inhibitory substrates. We selected ten substrate-inhibitors and substrate-non-inhibitors (Figure 5) with high structural and functional diversity in mind for pairwise inhibition testing. Each compound was used as substrate (horizontal lines in Figure 6) and inhibitor against all other substrates (vertical columns in Figure 6).

We found that some OCT1 substrates, such as naratriptan, sulpiride and sumatriptan, showed little to no inhibition of the transport of 9 of the 10 tested substrates. Interestingly, ASP^+^ led to more than 100% uptake inhibition of dobutamine compared with control, hinting towards OCT1-dependent efflux (181% ± 16 SEM). This finding was stable across six independent replications. Most strikingly among them, sumatriptan showed ‘negative inhibition’ (i.e., enhanced transport) of the transport of three well established OCT1 substrates, and while only minor transport enhancement was noted with ASP^+^ and berberine, strong transport enhancement was reproducibly found for dobutamine across all replications. Notably, those substrates with high inhibition of ASP^+^ and sumatriptan in the previous large-scale screening, represented by denatonium and dobutamine, were also among the substrates that were inhibited by many other substrates. On the opposite site of the spectrum were ASP^+^ and berberine, which showed relatively little inhibitability. Interestingly, some substrate-inhibitor combinations showed reciprocally similar results (e.g., dobutamine and berberine did inhibit each other comparably strongly), while other combinations showed an inhibitory effect in one direction, but not in the other (e.g., ASP^+^ inhibited sumatripan uptake but not vice versa). To further elucidate the interaction of dobutamine with sumatriptan, and of sulpiride with naratriptan, we tested the concentration-dependence of the interaction between these combinations.

### 2.5. Pairwise Concentration-Dependent Inhibition for Dobutamine and Sumatriptan

**Figure 7:**
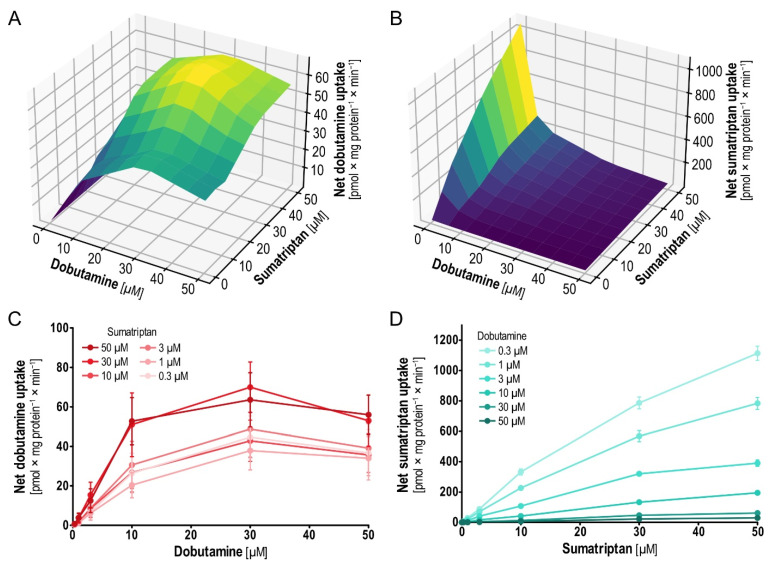
Concentration-dependent mutual inhibition of dobutamine and sumatriptan uptake. (**A**) Three-dimensional plot showing means of intracellular dobutamine concentration of three independent experiments and interpolations. (**B**) Three-dimensional plot showing means of intracellular sumatriptan concentration of three independent experiments. (**C**,**D**) Two-dimensional projections of (**A**,**B**) including means and SEM of three independent experiments.

When testing pairwise uptake inhibition with dobutamine and sumatriptan in a concentration-dependent manner (0.3–50 µM; uninhibited kinetics have already been published [9,16]), dobutamine showed strong inhibition of sumatriptan uptake. In contrast, sumatriptan showed no inhibition of dobutamine uptake (Figure 7). More than that, across the entire range of dobutamine concentrations, higher sumatriptan concentrations surprisingly increased dobutamine uptake, with the largest effects at 30 µM dobutamine and sumatriptan concentrations of 30–50 µM. Interestingly, the effect was higher at dobutamine concentrations below saturation, which suggests transporter involvement instead of off-target effects. Dobutamine uptake increased with increasing sumatriptan concentrations, so a plausible dose–effect relationship could be shown, also bolstering the hypothesis of more than a spurious phenomenon. In search of additional insights on potential interactions of dobutamine and sumatriptan in the OCT1 binding pocket, we performed virtual docking (using AutoDock Vina [26] and AlphaFold-generated OCT1 [27]). Virtual docking suggested merely identical binding sites (Figure S3), in line with noncompetitive non-allosteric inhibition.

The increased *V*_max_ of dobutamine uptake became apparent for the higher sumatriptan concentrations, while *K*_m_ remained unchanged (Table 3). In contrast to this, increasing dobutamine concentration inhibited sumatriptan uptake by reducing its *V*_max_.

Due to the interesting results for sumatriptan and dobutamine we also decided to test the combination of sumatriptan and the structurally dobutamine-resembling and biaromatic ractopamine. Ractopamine is also among the OCT1-inhibiting OCT1 substrates (Figure 5, Table 4). Results showed that sumatriptan did also enhance the uptake of ractopamine, whereas ractopamine also inhibited the uptake of sumatriptan by reducing its *V*_max_ (Figure S4). As dobutamine has structural properties of the neurotransmitter dopamine, and sumatriptan has structural properties of serotonin, we additionally investigated the hypothesis of serotonin-enhanced dopamine uptake in OCT1 (and structurally related OCT2 and OCT3). However, there was neither enhancement nor inhibition of dopamine uptake by addition of serotonin and vice versa (data not shown).

**Figure 8:**
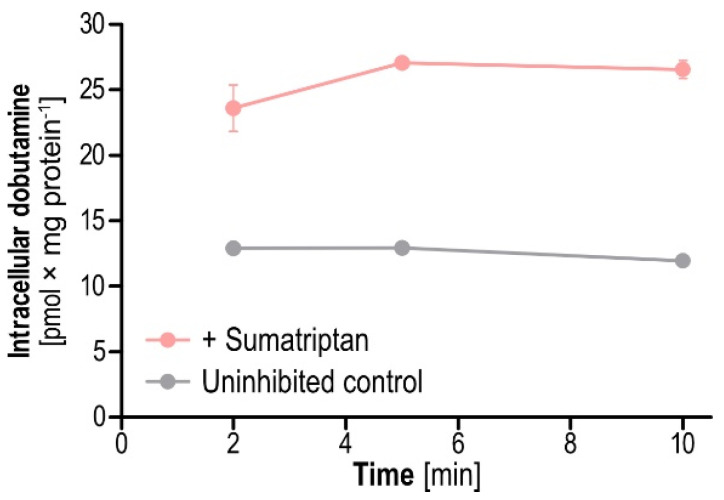
Dobutamine efflux with and without the addition of sumatriptan. Dobutamine efflux was reduced by the addition of sumatriptan, leading to increased intracellular dobutamine concentrations by about twofold. Results show means ± SEM of intracellular concentrations for three independent experiments, in which the efflux of 1 µM dobutamine from cells after 60 min preloading was inhibited for 2, 5 and 10 min by addition of 10 µM sumatriptan to the extracellular medium during the efflux period.

To follow up on the potential mechanisms behind the observed enhanced transport of dobutamine uptake by sumatriptan, we investigated sumatriptan-mediated inhibition of dobutamine efflux from preloaded cells. Results showed that 2, 5 and 10 min after addition of sumatriptan, efflux of preloaded dobutamine was reduced (Figure 8). Remaining intracellular dobutamine was increased by 2.0-, 2.1 and 2.5-fold compared to the non-inhibited control when sumatriptan was added to the extracellular buffer during the efflux period. This hints at OCT1-mediated efflux of dobutamine, inhibitable by sumatriptan, as one possible mechanism for enhanced intracellular accumulation.

### 2.6. Pairwise Concentration-Dependent Inhibition for Sulpiride and Naratriptan

**Figure 9:**
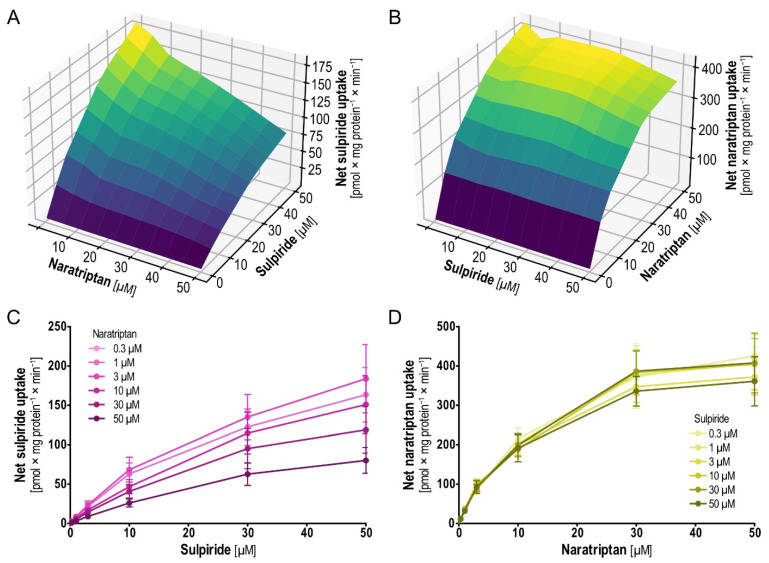
Concentration-dependent mutual inhibition of sulpiride and naratriptan uptake. (**A**) Three-dimensional plot showing means of intracellular sulpiride concentration of three independent experiments. (**B**) Three-dimensional plot showing means of intracellular naratriptan concentration of three independent experiments. (**C**,**D**) Two-dimensional projections of (**A**,**B**) including means and SEM of three independent experiments.

With the second pair that showed interesting results in the mutual inhibition testing, sulpiride and naratriptan, a picture different from that for dobutamine and sumatriptan emerged (Figure 9). Sulpiride uptake was increasingly inhibited with increasing concentrations of naratriptan (Figure 9A,C). On the other side and more surprising, naratriptan uptake was constant, irrespective of the presence of sulpiride at varying concentrations (Figure 9B,D).

Analysis of sulpiride uptake follows Michaelis–Menten kinetics [28]. Upon addition of naratriptan in concentrations of 0.3, 1, 3, 10, 30 and 50 µM the noncompetitive inhibition of sulpiride uptake through naratriptan was indicated by a reduction in the *V*_max_ (Table 4). Strikingly, no net change in the naratriptan uptake was observed when coincubating together with sulpiride. In summary, these findings once more point towards the complexity of possible interactions at solute carriers. Same as for dobutamine and sumatriptan, virtual docking suggested merely identical binding sites for sulpiride and naratriptan (Figure S5), in line with noncompetitive nonallosteric inhibition of sulpiride by naratriptan. For those interested in the structures of the main model substrates used in this work, including sulpiride, naratriptan, dobutamine, sumatriptan and ASP^+^, we refer to Figure S6.

## 3. Discussion

Overall, this study showed that most OCT1 substrates were only weak inhibitors. However, there were some remarkable exceptions. Explicitly, biaromatic β-agonistic compounds, such as fenoterol, dobutamine, ractopamine and ritodrine inhibited either of the model substrates quite strongly. Extending the list of well-established OCT1 model substrates to ten structurally diverse compounds for pairwise inhibition testing, interestingly asymmetric patterns of inhibition, non-inhibition and uptake enhancement could be found. Concentration-dependent analyses of selected compound pairs showed strongly enhanced uptake of dobutamine by sumatriptan. In addition, naratriptan inhibited sulpiride uptake noncompetitively but sulpiride did not inhibit naratriptan uptake at all. With regard to enhanced uptake of dobutamine by addition of sumatriptan, we could finally show that inhibition of dobutamine efflux could be one mechanism increasing net dobutamine uptake. In summary our study highlights that interactions with OCT1 should not be reduced to uptake and uptake inhibition, but might also include efflux-specific inhibition and potentially other mechanisms increasing net uptake. As they will, for sure interact with OCT1, known OCT1 substrates are interesting screening candidates to find more complex drug–transporter interactions. As OCT1 is an efficient serotonin transporter and OCT2 is an efficient serotonin and dopamine transporter [23,29], potential complex interactions of other OCT substrates with serotonin and dopamine could prove valuable for pharmacological interventions modulating peripheral blood serotonin concentrations.

### 3.1. OCT1 Substrates and Inhibitors

While OCT1 inhibition has been studied intensively, there is much less data on OCT1 transport [10,14,16]. In particular, only little data was available on the transport of the many compounds tested for OCT1 inhibition. To fill this void, we measured uptake of 165 compounds tested for inhibition as well (Table S1).

There have been several large-scale screenings for OCT1 inhibitors [11,17]. Here we aimed for enriching the test compounds with known OCT1 substrates, but also included previously tested compounds to check for interlaboratory consistency. The high consistency found was surprising, given the previously reported high inter-laboratory variability in transport data [30].

Regarding inhibitors, our results were in line with prior publications on general OCT1 inhibition (not focusing on substrates), where often highly lipophilic compounds that did not match the typical OCT1 substrate structure, were among the most efficient inhibitors e.g., spironolactone, verapamil and chlorprothixen [11,17,31,32]. Focusing on OCT1-substrates tested for OCT1 inhibition, a huge variation in inhibition could be observed, although one might have expected generally higher inhibition potential (due to competitive binding, Table 4). Those vast differences in inhibitory potential for different OCT1 substrates may suggest different binding sites, since slight differences in the affinity only provide insufficient justification for the results found. While screening data does not provide sufficient proof, the above-presented, published kinetic data of substrates tested as inhibitors (Table 4) showed that inhibition cannot solely be explained by competition.

As OCTs inhibition has been found to be in part dependent on the substrate used [3,33,34], in addition to ASP^+^, we tested a clinically relevant substrate, sumatriptan. While some compounds showed differences between inhibition of ASP^+^ and inhibition of sumatriptan, there was mainly consistency in inhibition of both, with slightly higher inhibitability of sumatriptan. This is in line with published data [33,34].

In contrast to some previous publications, we and others (Figure 4) have shown overall correlation of inhibition using different substrates, albeit with outliers. These indicate that the concept of a model substrate has its limitations. Many previous publications indicate existence of multiple binding sites for substrates and multiple mechanisms for inhibition [35,36,37,38]. Thus, it is unlikely that one model substrate really predicts all relevant interactions. For instance, a scaffold consistently associated with high inhibition was that of biaromatic β-agonists such as fenoterol and dobutamine. While this common scaffold already supported a structural explanation over an experimental artifact, equivalent results for a second model substrate and the concentration-dependent experiments on dobutamine and ractopamine inhibition of sumatriptan confirmed it. Interestingly, although high inhibition by β-agonistic substrate-inhibitors could be explained competitively (Table 2), inhibition was indeed noncompetitive (Figure 7 and Figure S4). Therefore, competitive substrate-inhibitors were rarely identified by our screening and the best substrate-inhibitors were not competitive. In other words, inhibitor screening is not well suited to finding OCT substrates.

### 3.2. Mutual Substrate Inhibition

**Figure 10:**
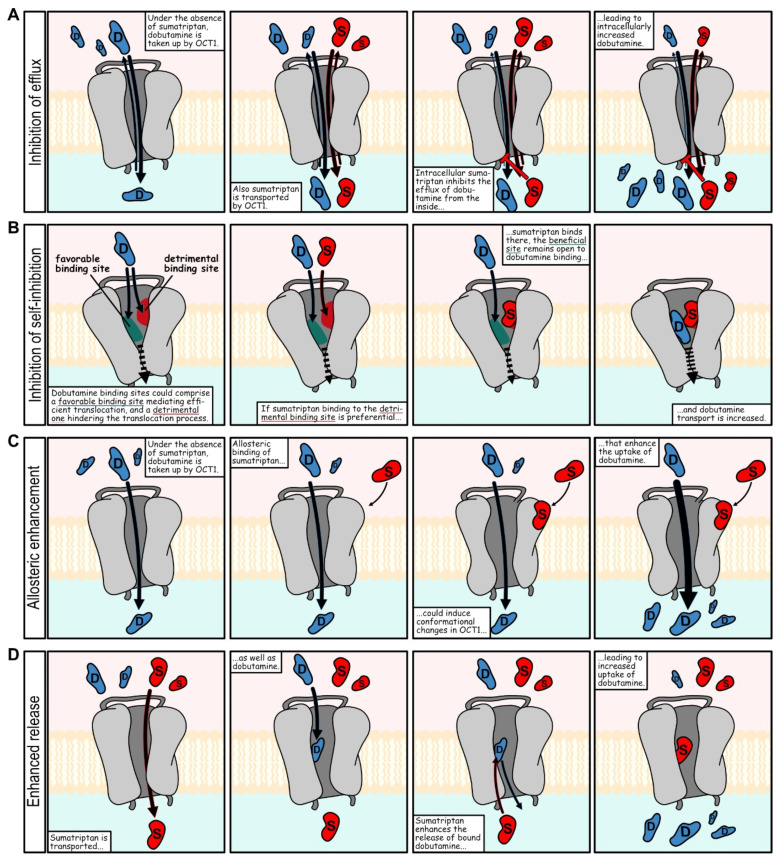
Potential mechanisms leading to increased intracellular dobutamine concentrations. Among them are (**A**) inhibition of dobutamine efflux by intracellular sumatriptan, (**B**) inhibition of dobutamine-self-inhibition by sumatriptan from the extracellular side, (**C**) allosteric enhancement, and (**D**) enhanced dobutamine release by sumatriptan.

Integrating the findings on OCT1 inhibition by OCT1 substrates, it was remarkable that only a few substrates showed relevant inhibition (Figure 5), given that each of them was expected to interfere competitively. Notably, these findings were based on a single concentration for uptake measurement and inhibition of two model substrates only. Although this point has already been addressed by further kinetic data on the inhibitor candidates tested (Table 2), we decided for the extension of the model substrates and pairwise inhibition screening. Within this pairwise inhibition set, inhibition as well as inhibitability varied largely. The observed inhibition above 100% for ASP^+^ and dobutamine could be explained by ASP^+^-mediated dobutamine efflux via OCT1. Surprisingly, the good inhibitors dobutamine and denatonium were among those that could be inhibited quite well, which speaks against a pure affinity-based explanation with a single binding site. Interestingly, naratriptan, sulpiride and sumatriptan were relatively weak inhibitors and most strikingly, sumatriptan even strongly enhanced dobutamine uptake. This effect was shown to be concentration dependent. Given that dobutamine also was an inhibitor of sumatriptan uptake, there are several explanations for how sumatriptan could improve dobutamine uptake. Possible scenarios include inhibition of efflux, inhibition of self-inhibition, allosteric and noncompetitive modulation as well as enhanced release [39] (Figure 10).

With inhibition of dobutamine efflux, sumatriptan would block the intracellular binding site for efflux transport of dobutamine. Inhibition of self-inhibition includes a scenario, in which sumatriptan blocks an unfavorable dobutamine binding site. Allosteric enhancement of dobutamine uptake, means sumatriptan binding enhances dobutamine uptake. The effect of increased uptake might have been discovered in earlier inhibition screenings, where some compounds increased uptake of model substrates instead of inhibiting it [11,17,34]. In contrast to those enhancers that were secondary findings in prior inhibition screenings, the effects of the combination of dobutamine and sumatriptan were relatively large and demonstrably concentration dependent (Figure 7). Our concentration-dependent experiments on dobutamine and sumatriptan were motivated by our screening results, which predicted the later concentration-dependent results quite well. Interactions at OCT1 apparently exceed the classical competitive and noncompetitive inhibition from extracellular sources and might include efflux inhibition, as shown by the example of sumatriptan-mediated enhancement of dobutamine (Figure 8). Net uptake results might better be viewed as influenced by the sum of several processes taking place simultaneously, all of them possibly inhibitable and probably enhanceable. On a side note, reduced efflux of preloaded dobutamine, when subsequently adding the OCT1 substrate sumatriptan might hint at some difficulties for trans-stimulation and counterflow approaches. In transstimulation and counterflow assays, addition of a substrate is supposed to facilitate OCT-mediated efflux of a preloaded model substrate. Substrates that also inhibit efflux of the model substrate could remain unnoticed when using these approaches for screening.

### 3.3. Enhancing Transporter Function in Therapeutics

Enhancing facilitated transport has been of interest for several diseases, most prominently with cystic fibrosis transmembrane conductance regulator (CFTR) and cystic fibrosis but also with the glutamate transporters and amyotrophic lateral sclerosis [40,41,42]. While from a clinically applied vantage point, OCT1 currently does not rank among the transporters that are the most viable drug targets, OCT1 specifically and OCTs in general have the advantage of polyspecificity. With the hypothesis that there is a higher probability of reaching transport-enhancing target interaction with substances that interact with the target at all, a transporter with a high number of known and structurally diverse substrates, is of course a good candidate for testing. When interested in clinical applicability of OCT enhancement, the focus for further testing should be guided towards enhanced transport of endogenous OCT substrates.

Compared with agonists and inhibitors, enhancers play no significant role in current pharmacotherapy, although they would be an interesting option. Interestingly, enhancement has been discussed in connection with transporters, for example with the serotonin reuptake transporter (SERT), where for an allosteric binding site, a possible modulation of the substrate binding site was discussed [39]. Based on crystallographic results, the authors speculate, that allosteric binding by small molecules could lead to structural changes resulting in modified transport capacity, from inhibition up to enhancement [39].

Given that transporters remain among the most underrepresented target structures relative to their abundance, transporters might be the sleeping giant in drug discovery. With the G-protein-coupled receptor always being investigated with regard to inhibition as well as agonism, and transporters being essential for homeostasis, it is astonishing that transport has primarily been investigated with the intent to suppress it.

Indications of enhancement of OCT1 transport by chemical modulators can be found in the literature for more than 10 years but have, thus far, not received much attention [43,44]. This study showed that solute carrier-mediated uptake can be enhanced by inhibiting their efflux. For substrates, in addition to interactions due to different influx binding sites, there might also be interactions caused by efflux binding sites. This makes substrates not the boring competitive inhibitors one might expect, but the flamboyant multifaceted stars of solute carrier kinetics, that might also work as enhancers of membrane transport.

## 4. Materials and Methods

### 4.1. In Vitro Inhibition Experiments

Inhibition experiments with model substrates ASP^+^ and sumatriptan (both Sigma-Aldrich, Taufkirchen, Germany) were conducted with HEK293 cells, plated as 300,000 cells per well in poly-D-lysine (Sigma-Aldrich, Taufkirchen, Germany) pre-coated 24-well plates (Greiner Bio-One, Kremsmünster, Austria) 48 h prior to the transport experiment. For each measurement, empty vector (EV)-transfected cells were used to quantify unspecific uptake of the model substrate, and OCT1-transfected cells were used to quantify the uptake of model substrate under non-inhibitory conditions. For the inhibition experiment, cells were washed with 37 °C HBSS (Thermo Fisher Scientific, Darmstadt, Germany) supplemented with 10 mM HEPES pH 7.4 (Sigma-Aldrich, Taufkirchen, Germany)—hereafter termed HBSS+—once. Next, cells were incubated with 2 µM of the model substrate with and without 20 µM of the compound-in-question in HBSS+ for 5 min. After that, the reaction was stopped, and cells were washed twice with 4 °C HBSS+. Cells were lysed in 80% acetonitrile (LGC Standards, Wesel, Germany) and incubated for 10 min on a 3D platform shaker (Heidolph, Schwabach, Germany) before being transferred into a 96-well plate for fluorescence measurement (ASP^+^) or quantification by HPLC-MS/MS (see below). Fluorescence of intracellularly accumulated ASP^+^ was performed using a Tecan Ultra (Tecan, Crailsheim, Germany) multiplate reader with an excitation wavelength of 482 nm and an emission wavelength of 612 nm. All inhibition experiments were performed at least three times independently, and each fluorescence quantification of model substrate uptake inhibition was performed with technical duplicates. Percentage of OCT1 inhibition was calculated according to the following equation:

$$\%\mathrm{OCT}1\;\mathrm{inhibition}=100\%-\frac{\left(\left[{\mathrm{uptake}}_{\mathrm{inhibited}}\right]-\left[{\mathrm{uptake}}_{\mathrm{EV}\;\mathrm{control}}\right]\right)\times 100}{\left(\left[{\mathrm{uptake}}_{\mathrm{uninhibited}}\right]-\left[{\mathrm{uptake}}_{\mathrm{EV}\;\mathrm{control}}\right]\right)}$$

As an indicator of screening quality, Z′ was calculated for measurements and reached an average value of 0.75. Pre-incubation with the inhibitor had been evaluated for 20 selected compounds in advance but did not show any significant effect on inhibition. Linearity of uptake within the incubation period was not tested for each compound individually, in agreement with the general methodology of screening approaches, but linearity within the first 5 min of incubation is very much in line with previously tested substrates [5,9,12,45]. All used compounds were purchased from renowned manufacturers with purities of at least 95%.

### 4.2. In Vitro Uptake Experiments

Transport experiments were in principle performed as described earlier [15,46]. Identical to inhibition experiments, 300,000 HEK293 cells stably transfected with the empty vector pcDNA5 or to overexpress human OCT1 were plated per well in poly-D-lysine pre-coated 24-well plates 48 h prior to the transport experiment. For uptake experiments, cells were washed once with 37 °C HBSS (Thermo Fisher Scientific, Darmstadt, Germany) supplemented with 10 mM HEPES pH 7.4 (Sigma-Aldrich, Taufkirchen, Germany). Empty vector and OCT1-transfected cells were incubated with 2.5 µM of the respective drug in HBSS+ at 37 °C. After two minutes, cells were washed with ice-cold HBSS+ twice and lysed with 500 µL 80% (*v*/*v*) acetonitrile (LGC Standards, Wesel, Germany) including an internal standard. Intracellular accumulation of tested substances was measured by LC-MS/MS and calculated as fold-change of the uptake by dividing uptake into OCT1 overexpressing cells through uptake into EV cell.

To test the sumatriptan-mediated inhibition of dobutamine efflux, cells were preloaded with 1 µM dobutamine in HBSS+ after the initial washing step with 37 °C HBSS+. After 60 min, cells were washed twice with 37 °C HBSS+ and incubated with HBSS+ with or without 10 µM sumatriptan in HBSS+ for 2, 5 or 10 min. Cells were then washed and lysed with 500 µL 80% (*v*/*v*) acetonitrile and the remaining intracellular dobutamine was measured by LC-MS/MS.

Normalization of cell numbers was performed by measuring RIPA-lysed total protein via a standard bicinchoninic acid assay. All used compounds were purchased from renowned manufacturers with purities of at least 95%.

### 4.3. HPLC-MS/MS Concentration Analyses

The cellular accumulation of compounds for OCT1 uptake analyses were quantified by HPLC-MS/MS using a Shimadzu Nexera HPLC system (autosampler SIL-30AC, column oven CTO-20AC, pump LC-30AD pump, controller CBM-20A, all from Shimadzu, Kyoto, Japan). Separation was achieved on a Brownlee SPP RP-Amide column (4.6 × 100 mm inner dimension with 2.7 µm particle size) with a C18 pre-column. Reversed-phase chromatography was achieved with an aqueous mobile phase containing 0.1% (*v*/*v*) formic acid and organic additive (acetonitrile:methanol (6:1)) ranging von 3 to 50% (*v*/*v*) with a flow rate of 0.3 or 0.4 mL/min and a column temperature of 40 °C. Investigated compounds were detection with an API 4000 tandem mass spectrometer (AB SCIEX, Darmstadt, Germany) operating in MRM mode. Analyte peaks were integrated and quantified using the Analyst software (Version 1.6.2, AB SCIEX, Darmstadt, Germany).

### 4.4. Calculations and Software

Cellular net uptake was calculated as difference between uptake in HEK293 cells stably transfected to overexpress OCT1 and empty vector-transfected control. Kinetic parameters were calculated using GraphPad Prism 5 (v 5.01; GraphPad Software, San Diego, CA, USA). Virtual docking was performed using AutoDock Vina [26] and AlphaFold-generated OCT1 [27]. The database was curated in DataWarrior (v 5.5.0; Idorsia Pharmaceuticals Ltd., Allschwil, Switzerland) and contained data made available through several publications [11,14,16,17]. LogD values for investigated compounds were calculated using the cxcalc tool from the InstantJChem package (ChemAxon, Budapest, Hungary). Three-dimensional wave plots were generated using mpl_toolkits, SciPy, and Matplotlib.

## Figures and Tables

**Table 1:** Chemical descriptors for compounds (non-)inhibiting OCT1-mediated ASP^+^ uptake grouped by transport.

		Non-Inhibitors/Weak Inhibitors (≤40% Inhibition)	Inhibitors (>40% Inhibition)
Good substrates (ratio ≥ 3)	N	65	5
	MW	245 (109–369)	296 (285–303)
	LogD_7.4_	−1.2 (−4.3–1.1)	0.5 (−0.6–1.2)
	TPSA	63.5 (0–175.8)	72.8 (52.9–93.0)
	Charge_7.4_	0.98 (0–2)	1.00 (1–1)
	Ring count	1.7 (0–5)	2.6 (2–5)
	H bond donors	2.2 (0–4)	3.8 (2–5)
Poor substrates (3 > ratio ≥ 1.5)	N	42	8
	MW	213 (61–404)	315 (179–587)
	LogD_7.4_	−0.7 (−4.7–3.1)	0.6 (−0.6–1.6)
	TPSA	50.8 (12.0–105.5)	53.9 (21.3–114.2)
	Charge_7.4_	0.90 (0–1)	1.00 (1–1)
	Ring count	1.6 (0–5)	2.3 (1–7)
	H bond donors	1.9 (0–4)	1.3 (1–2)
Non-substrates (ratio < 1.5)	N	133	20
	MW	233 (75–645)	351 (207–506)
	LogD_7.4_	−0.8 (−7.4–6.0)	1.5 (−2.5–3.6)
	TPSA	61.7 (6.5–169.0)	50.8 (3.2–177.6)
	Charge_7.4_	0.55 (−1–3)	1.1 (1–2)
	Ring count	1.7 (0–6)	3.1 (2–5)
	H bond donors	1.7 (0–7)	1.4 (0–6)

**Table 2:** Expected and observed inhibition kinetics for selected OCT1 substrates.

Substrate Inhibitor Candidates	Apparent *K*_m_ [µM]	ASP^+^ Inhibition in Our Assay (in %)	Inhibition ≥ 50% Expected	Inhibition ≥ 50% Observed
Benzyltriethylammonium	38.6 [16]	−5	No	No
Denatonium	12.6 [16]	12	Yes	No
Edrophonium	26.4 [16]	−3	No	No
Famotidine	35.7 [16]	−20	No	No
Fenoterol	1.2 [12]	68	Yes	Yes
Fenpiverinium	5.9 [16]	9	Yes	No
Guanfacine	8.6 [16]	24	Yes	No
Ipratropium	12.3	14	Yes	No
mIBG	15.9 [16]	7	Yes	No
Milnacipran	2.26 [16]	39	Yes	No
Prenalterol	13.3 [16]	−3	Yes	No
Ractopamine	2.1 [16]	56	Yes	Yes
Ritodrine	1.7 [16]	67	Yes	Yes
Sumatriptan	42.6 [9]	−7	No	No

The model substrate ASP^+^ (*K*_m_ = 9.2 µM) [11] was used in a concentration significantly below its *K*_m_ and inhibitors with concentrations of 20 µM. Index values indicating reference number.

**Table 3:** Analysis of OCT1 dobutamine and sumatriptan uptake following Michaelis–Menten kinetics.

Substrate	Inhibitor	Inhibitor [µM]	*K*_m_ [µM]		*V*_max_ [pmol × min^−1^ × mg Protein^−1^]	
Dobutamine	Sumatriptan	0.3	11.1	(±8.5)	51.9	(±12.9)
		1	13.9	(±10.4)	48.2	(±12.7)
		3	9.5	(±7.6)	54.5	(±13.5)
		10	9.6	(±6.9)	48.8	(±10.9)
		30	6.8	(±4.3)	73.1	(±12.9)
		50	7.2	(±4.0)	72.8	(±11.6)
Sumatriptan	Dobutamine	0.3	79.6	(±17.6)	2885	(±428)
		1	75.5	(±20.0)	1974	(±346)
		3	57.4	(±14.7)	863	(±133)
		10	181.6	(±111.2)	909	(±453)
		30	118.9	(±90.0)	213	(±120)
		50	111.9	(±75.6)	98	(±49)

**Table 4:** Analysis of OCT1 sulpiride and naratriptan uptake following Michaelis–Menten kinetics.

Substrate	Inhibitor	Inhibitor [µM]	*K*_m_ [µM]		*V*_max_ [pmol × min^−1^ × mg Protein^−1^]	
Sulpiride	Naratriptan	0.3	40.3	(±29.4)	323	(±129)
		1	35.8	(±23.0)	277	(±90)
		3	40.0	(±29.8)	327	(±129)
		10	54.7	(±48.4)	318	(±167)
		30	41.3	(±34.5)	220	(±99)
		50	47.9	(±35.9)	159	(±67)
Naratriptan	Sulpiride	0.3	16.1	(±6.5)	565	(±85)
		1	15.7	(±6.1)	548	(±78)
		3	13.0	(±3.8)	478	(±49)
		10	16.3	(±6.3)	554	(±80)
		30	16.1	(±6.5)	558	(±84)
		50	13.1	(±4.8)	465	(±59)

Data provided as means of at least three independent experiments ± SEM.

## Data Availability

All data newly published within this article can be found in the Supplementary Materials.

## Supplementary Materials

The following supporting information can be downloaded at: https://www.mdpi.com/article/10.3390/ijms23042007/s1.

## References

[1] Nies A.T., Koepsell H., Winter S., Burk O., Klein K., Kerb R., Zanger U.M., Keppler D., Schwab M., Schaeffeler E. (2009). Expression of organic cation transporters OCT1 (SLC22A1) and OCT3 (SLC22A3) is affected by genetic factors and cholestasis in human liver. Hepatology.

[2] Hilgendorf C., Ahlin G., Seithel A., Artursson P., Ungell A.-L., Karlsson J. (2007). Expression of thirty-six drug transporter genes in human intestine, liver, kidney, and organotypic cell lines. Drug Metab. Dispos..

[3] Koepsell H. (2020). Organic cation transporters in health and disease. Pharmacol. Rev..

[4] Suhre K., Shin S.Y., Petersen A.K., Mohney R.P., Meredith D., Wagele B., Altmaier E., Gram C., Deloukas P., Erdmann J. (2011). Human metabolic individuality in biomedical and pharmaceutical research. Nature.

[5] Meyer M.J., Seitz T., Brockmöller J., Tzvetkov M.V. (2017). Effects of genetic polymorphisms on the OCT1 and OCT2-mediated uptake of ranitidine. PLoS ONE.

[6] Liang X., Yee S.W., Chien H.-C., Chen E.C., Luo Q., Zou L., Piao M., Mifune A., Chen L., Calvert M.E. (2018). Organic cation transporter 1 (OCT1) modulates multiple cardiometabolic traits through effects on hepatic thiamine content. PLoS Biol..

[7] Shu Y., Brown C., Castro R., Shi R., Lin E., Owen R., Sheardown S., Yue L., Burchard E., Brett C. (2008). Effect of genetic variation in the organic cation transporter 1, OCT1, on metformin pharmacokinetics. Clin. Pharm..

[8] Venkatasubramanian R., Fukuda T., Niu J., Mizuno T., Chidambaran V., A Vinks A., Sadhasivam S. (2014). ABCC3 and OCT1 genotypes influence pharmacokinetics of morphine in children. Pharmacogenomics.

[9] Matthaei J., Kuron D., Faltraco F., Knoch T., Pereira J.N.D.S., Abu Abed M., Prukop T., Brockmöller J., Tzvetkov M.V. (2016). OCT1 mediates hepatic uptake of sumatriptan and loss-of-function OCT1 polymorphisms affect sumatriptan pharmacokinetics. Clin. Pharm..

[10] Hendrickx R., Johansson J.G., Lohmann C., Jenvert R.-M., Blomgren A., Börjesson L., Gustavsson L. (2013). Identification of novel substrates and structure-activity relationship of cellular uptake mediated by human organic cation transporters 1 and 2. J. Med. Chem..

[11] Ahlin G., Karlsson J., Pedersen J.M., Gustavsson L., Larsson R., Matsson P., Norinder U., Bergström C., Artursson P. (2008). Structural requirements for drug inhibition of the liver specific human organic cation transport protein 1. J. Med. Chem..

[12] Tzvetkov M.V., Matthaei J., Pojar S., Faltraco F., Vogler S., Prukop T., Seitz T., Brockmöller J. (2018). Increased systemic exposure and stronger cardiovascular and metabolic adverse reactions to fenoterol in individuals with heritable OCT1 deficiency. Clin. Pharm..

[13] Longo N. (2016). Primary Carnitine Deficiency and Newborn Screening for Disorders of the Carnitine Cycle. Ann. Nutr. Metab..

[14] Meyer M.J., Neumann V.E., Friesacher H.R., Zdrazil B., Brockmöller J., Tzvetkov M.V. (2019). Opioids as substrates and inhibitors of the genetically highly variable organic cation transporter OCT1. J. Med. Chem..

[15] Jensen O., Rafehi M., Tzvetkov M.V., Brockmoller J. (2020). Stereoselective cell uptake of adrenergic agonists and antagonists by organic cation transporters. Biochem. Pharm..

[16] Jensen O., Brockmoller J., Ducker C. (2021). Identification of Novel High-Affinity Substrates of OCT1 Using Machine Learning-Guided Virtual Screening and Experimental Validation. J. Med. Chem..

[17] Chen E.C., Khuri N., Liang X., Stecula A., Chien H.-C., Yee S.W., Huang Y., Sali A., Giacomini K.M. (2017). Discovery of Competitive and Noncompetitive Ligands of the Organic Cation Transporter 1 (OCT1; SLC22A1). J. Med. Chem..

[18] Koepsell H. (2019). Multiple binding sites in organic cation transporters require sophisticated procedures to identify interactions of novel drugs. Biol. Chem..

[19] Koepsell H. (2021). Update on drug-drug interaction at organic cation transporters: Mechanisms, clinical impact, and proposal for advanced in vitro testing. Expert Opin. Drug Metab. Toxicol..

[20] Keller T., Gorboulev V., Mueller T.D., Dotsch V., Bernhard F., Koepsell H. (2019). Rat Organic Cation Transporter 1 Contains Three Binding Sites for Substrate 1-Methyl-4-phenylpyridinium per Monomer. Mol. Pharm..

[21] Gebauer L., Arul Murugan N., Jensen O., Brockmoller J., Rafehi M. (2021). Molecular basis for stereoselective transport of fenoterol by the organic cation transporters 1 and 2. Biochem. Pharm..

[22] Meyer M.J., Tuerkova A., Römer S., Wenzel C., Seitz T., Gaedcke J., Oswald S., Brockmöller J., Zdrazil B., Tzvetkov M.V. (2020). Differences in Metformin and Thiamine Uptake between Human and Mouse Organic Cation Transporter 1: Structural Determinants and Potential Consequences for Intrahepatic Concentrations. Drug Metab. Dispos..

[23] Gebauer L., Jensen O., Neif M., Brockmoller J., Ducker C. (2021). Overlap and Specificity in the Substrate Spectra of Human Monoamine Transporters and Organic Cation Transporters 1, 2, and 3. Int. J. Mol. Sci..

[24] Minuesa G., Volk C., Molina-Arcas M., Gorboulev V., Erkizia I., Arndt P., Clotet B., Pastor-Anglada M., Koepsell H., Martinez-Picado J. (2009). Transport of lamivudine [(-)-beta-L-2’,3’-dideoxy-3’-thiacytidine] and high-affinity interaction of nucleoside reverse transcriptase inhibitors with human organic cation transporters 1, 2, and 3. J. Pharm. Exp..

[25] Vivian D., Polli J.E. (2014). Mechanistic interpretation of conventional Michaelis-Menten parameters in a transporter system. J. Eur. Fed. Pharm. Sci..

[26] Trott O., Olson A.J. (2010). AutoDock Vina: Improving the speed and accuracy of docking with a new scoring function, efficient optimization, and multithreading. J. Comput. Chem..

[27] Jumper J., Evans R., Pritzel A., Green T., Figurnov M., Ronneberger O., Tunyasuvunakool K., Bates R., Žídek A., Potapenko A. (2021). Highly accurate protein structure prediction with AlphaFold. Nature.

[28] Dos Santos Pereira J.N., Tadjerpisheh S., Abu Abed M., Saadatmand A.R., Weksler B., Romero I., Couraud P.-O., Brockmöller J., Tzvetkov M.V. (2014). The poorly membrane permeable antipsychotic drugs amisulpride and sulpiride are substrates of the organic cation transporters from the SLC22 family. AAPS J..

[29] Amphoux A., Vialou V., Drescher E., Brüss M., La Cour C.M., Rochat C., Millan M.J., Giros B., Bönisch H., Gautron S. (2006). Differential pharmacological in vitro properties of organic cation transporters and regional distribution in rat brain. Neuropharmacology.

[30] Ekins S., Polli J.E., Swaan P.W., Wright S.H. (2012). Computational modeling to accelerate the identification of substrates and inhibitors for transporters that affect drug disposition. Clin. Pharm..

[31] Morse B.L., Kolur A., Hudson L.R., Hogan A.T., Chen L.H., Brackman R.M., Sawada G.A., Fallon J.K., Smith P.C., Hillgren K.M. (2020). Pharmacokinetics of Organic Cation Transporter 1 (OCT1) Substrates in Oct1/2 Knockout Mice and Species Difference in Hepatic OCT1-Mediated Uptake. Drug Metab. Dispos..

[32] Cho S.K., Kim C.O., Park E.S., Chung J.-Y. (2014). Verapamil decreases the glucose-lowering effect of metformin in healthy volunteers. Br. J. Clin. Pharm..

[33] Sandoval P.J., Zorn K.M., Clark A.M., Ekins S., Wright S.H. (2018). Assessment of Substrate-Dependent Ligand Interactions at the Organic Cation Transporter OCT2 Using Six Model Substrates. Mol. Pharm..

[34] Hacker K., Maas R., Kornhuber J., Fromm M.F., Zolk O. (2015). Substrate-Dependent Inhibition of the Human Organic Cation Transporter OCT2: A Comparison of Metformin with Experimental Substrates. PLoS ONE.

[35] Popp C., Gorboulev V., Müller T.D., Gorbunov D., Shatskaya N., Koepsell H. (2005). Amino acids critical for substrate affinity of rat organic cation transporter 1 line the substrate binding region in a model derived from the tertiary structure of lactose permease. Mol. Pharm..

[36] Volk C., Gorboulev V., Kotzsch A., Muller T.D., Koepsell H. (2009). Five amino acids in the innermost cavity of the substrate binding cleft of organic cation transporter 1 interact with extracellular and intracellular corticosterone. Mol. Pharm..

[37] Gorboulev V., Shatskaya N., Volk C., Koepsell H. (2005). Subtype-specific affinity for corticosterone of rat organic cation transporters rOCT1 and rOCT2 depends on three amino acids within the substrate binding region. Mol. Pharm..

[38] Gorboulev V., Volk C., Arndt P., Akhoundova A., Koepsell H. (1999). Selectivity of the polyspecific cation transporter rOCT1 is changed by mutation of aspartate 475 to glutamate. Mol. Pharm..

[39] Coleman J.A., Green E.M., Gouaux E. (2016). X-ray structures and mechanism of the human serotonin transporter. Nature.

[40] Van Goor F., Hadida S., Grootenhuis P.D.J., Burton B., Cao D., Neuberger T., Turnbull A., Singh A., Joubran J., Hazlewood A. (2009). Rescue of CF airway epithelial cell function in vitro by a CFTR potentiator, VX-770. Proc. Natl. Acad. Sci. USA.

[41] Abebe B.T., Weiss M., Modess C., Tadken T., Wegner D., Meyer M.J., Schwantes U., Neumeister C., Scheuch E., Schulz H. (2020). Pharmacokinetic Drug-Drug Interactions Between Trospium Chloride and Ranitidine Substrates of Organic Cation Transporters in Healthy Human Subjects. J. Clin. Pharmacol..

[42] Liu F., Zhang Z., Levit A., Levring J., Touhara K.K., Shoichet B.K., Chen J. (2019). Structural identification of a hotspot on CFTR for potentiation. Science.

[43] Amphoux A., Millan M.J., Cordi A., Bönisch H., Vialou V., la Cour C.M., Dupuis D.S., Giros B., Gautron S. (2010). Inhibitory and facilitory actions of isocyanine derivatives at human and rat organic cation transporters 1, 2 and 3: A comparison to human alpha 1- and alpha 2-adrenoceptor subtypes. Eur. J. Pharmacol..

[44] Gründemann D., Koschker A.-C., Haag C., Honold C., Zimmermann T., Schömig E. (2002). Activation of the extraneuronal monoamine transporter (EMT) from rat expressed in 293 cells. Br. J. Pharm..

[45] Jensen O., Rafehi M., Gebauer L., Brockmöller J. (2021). Cellular Uptake of Psychostimulants—Are High- and Low-Affinity Organic Cation Transporters Drug Traffickers?. Front. Pharmacol..

[46] Jensen O., Matthaei J., Blome F., Schwab M., Tzvetkov M.V., Brockmöller J. (2019). Variability and heritability of thiamine pharmacokinetics with focus on OCT1 effects on membrane transport and pharmacokinetics in humans. Clin. Pharm..

