# Supplementary material for: Relationships between Inhibition, Transport and Enhanced Transport via the Organic Cation Transporter 1
Source: Int J Mol Sci. 2022 Feb 11;23(4):2007. doi: 10.3390/ijms23042007 (PMC8878159; doi:10.3390/ijms23042007)
Supplement: Supplementary file 1 [file ijms-23-02007-s001.zip › ijms-1586525-supplementary.pdf]

Relationships between Inhibition, Transport and Enhanced Transport via the Organic Cation Transporter 1

Ole Jensen, Lukas Gebauer, Jürgen Brockmöller, and Christof Dücker

Institute of Clinical Pharmacology, University Medical Center Göttingen, D-37075 Göttingen, Germany

Supplementary Material

Figure S1: Own and previously published substrate uptake ratios in OCT1 overexpressing cells over empty vector cells

Figure S2: Percentage inhibition of OCT1-mediated ASP^+ transport by various compounds

Figure S3: Virtual docking of dobutamine and sumatriptan inside the binding cleft of OCT1

Figure S4: Concentration-dependent mutual inhibition of ractopamine and sumatriptan uptake

Figure S5: Virtual docking of sulpiride and naratriptan inside the binding cleft of OCT1

Figure S6: Structures of the main model substrates used in this study

Table S1: Newly provided transport and inhibition data

Table S2: OCT1 substrates with an uptake ratio ≥ 3

Table S3: Screened OCT1 inhibitors with an ASP^+ uptake inhibition ≥ 50% and top five "negatively inhibiting" drugs

Table S4: Chemical descriptors for compounds (non-)inhibiting OCT1-mediated sumatriptan uptake grouped by transport

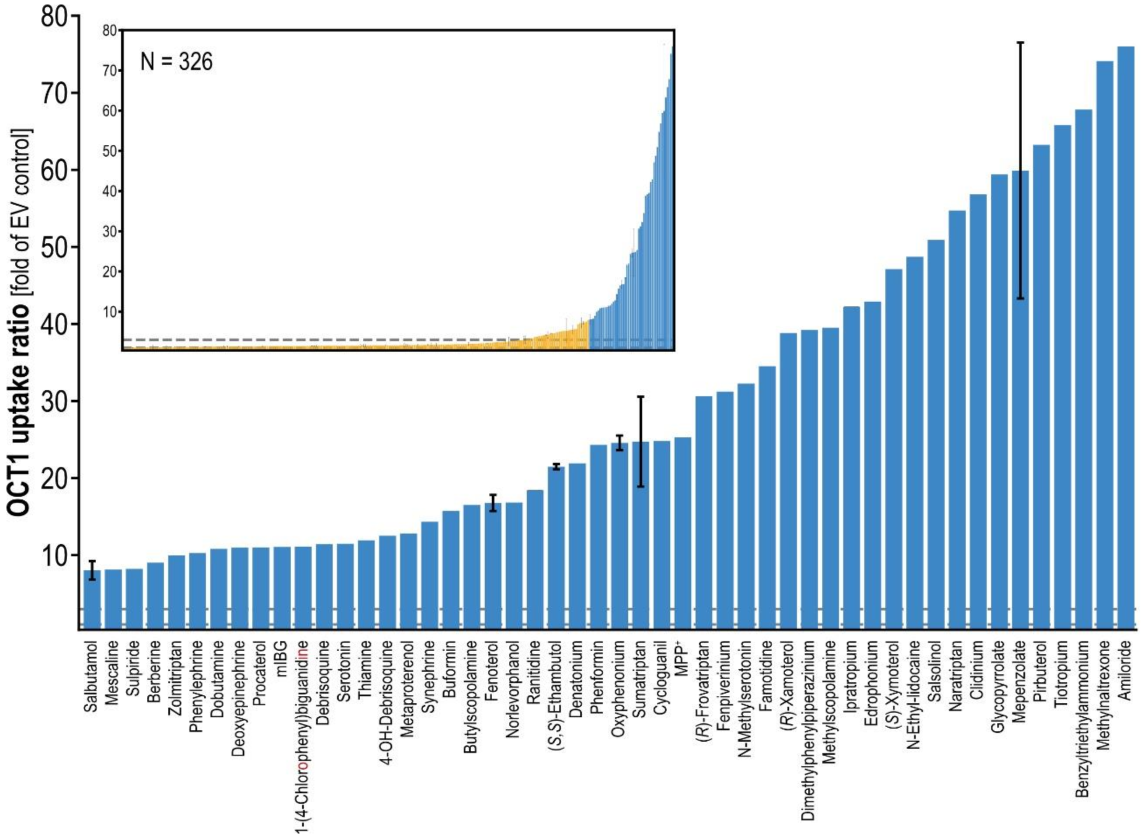

Figure S1: Own and previously published substrate uptake ratios in OCT1 overexpressing cells over empty vector cells. Error bars indicate standard error of the mean between publications. Large plot showing the top 50 substrates, miniature plot showing the overview of all 326 tested compounds. The upper dashed line indicates the uptake ratio of 3 considered by the authors as a reasonable cutoff for relevant transport.

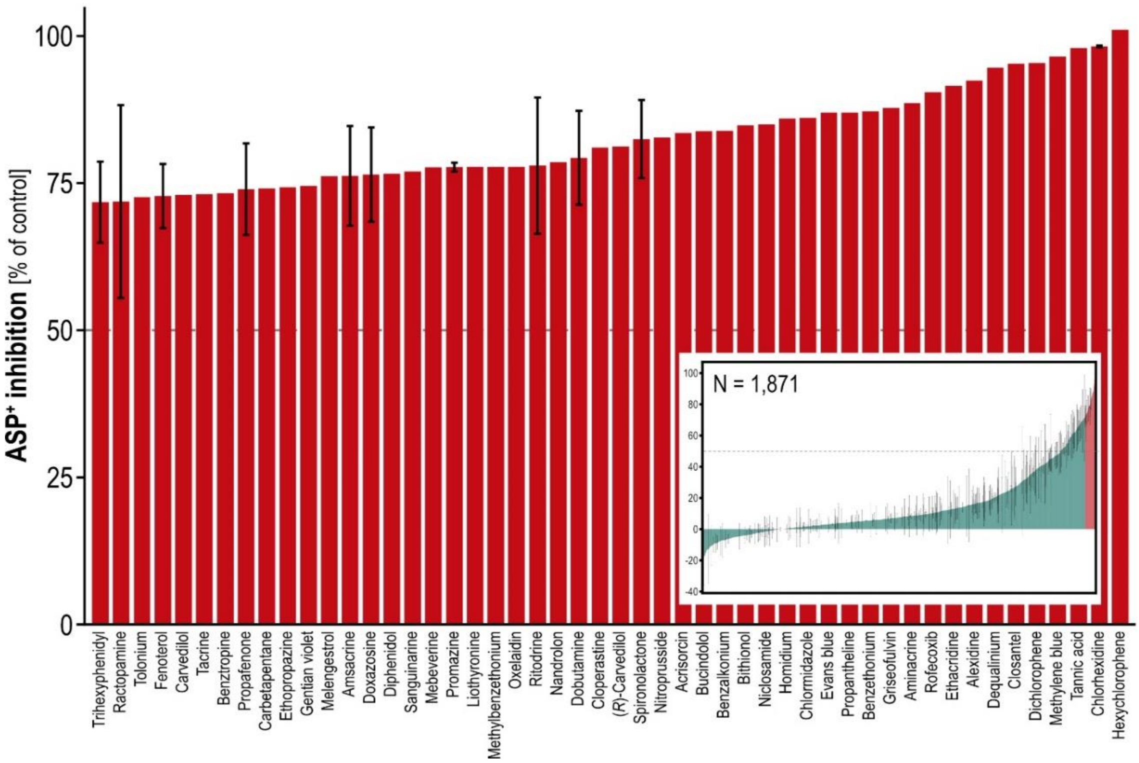

Figure S2: Percentage inhibition of OCT1-mediated ASP⁺ transport by various compounds. Own data integrated into previously published data, error bars indicate standard error of the mean between publications. Large plot showing the top 50 inhibitors, miniature plot showing the overview of all 1,871 tested compounds.

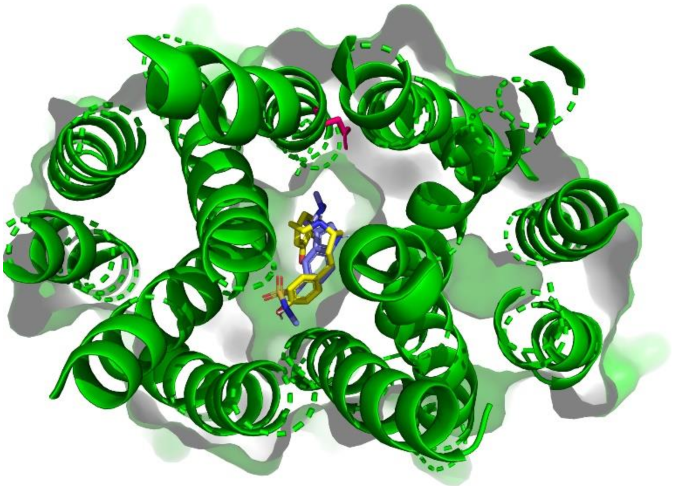

Figure S3: Virtual docking of dobutamine (yellow) and sumatriptan (purple) inside the binding cleft of OCT1 (green). The anionic amino acid Asp474 is highlighted in pink.

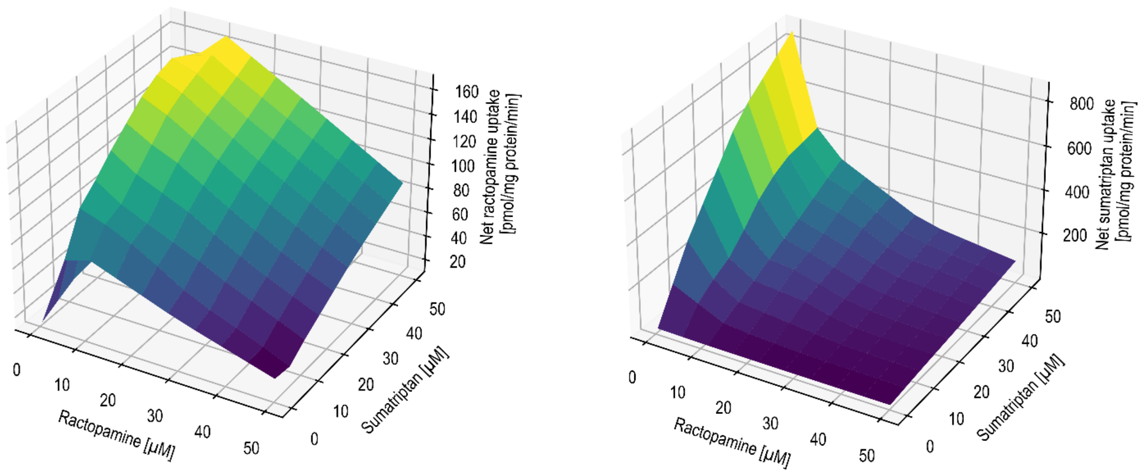

Figure S4: Concentration-dependent mutual inhibition of ractopamine and sumatriptan uptake. (A) Three-dimensional plot showing means of intracellular ractopamine concentration of three independent experiments and interpolations. (B) Three-dimensional plot showing means of intracellular sumatriptan concentration of three independent experiments.

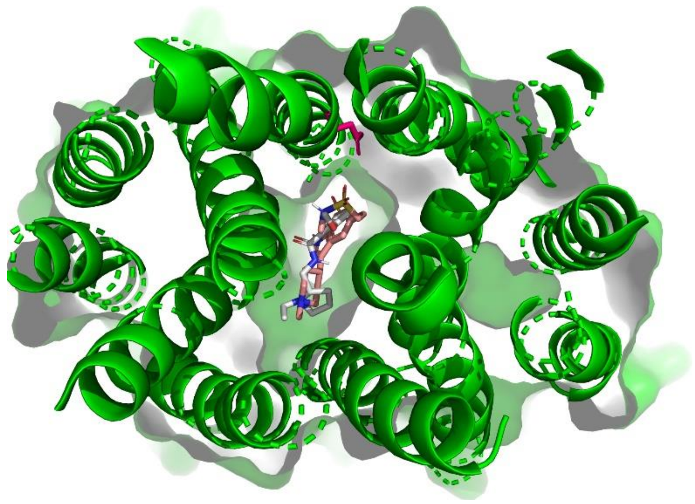

Figure S5: Virtual docking of sulpiride (white) and naratriptan (salmon) inside the binding cleft of OCT1 (green). The anionic amino acid Asp474 is highlighted in pink.

ASP+

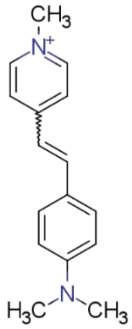

Dobutamine

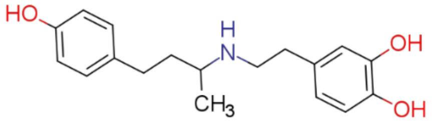

Naratriptan

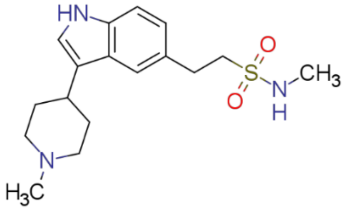

Sulpiride

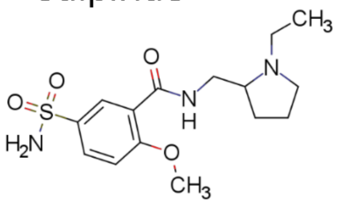

Sumatriptan

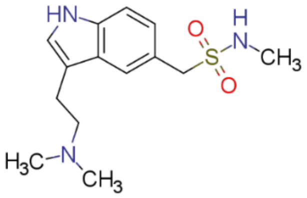

Figure S6: Structures of the main model substrates used in this study.

Table S1: Newly provided transport and inhibition data.

Drug	SMILES	Inhibition of ASP uptake [%]	Inhibition of sumatriptan uptake [%]	Uptake ratio [OCT1 / EV] from this publication	Uptake ratio [OCT1 / EV], prior data from our lab
(-)-adrenaline	CNC[C@H](O)c1ccc(O)c(O)c1	3			1.97
(-)-alpha-methylnorepinephrine	CC(N)C(O)c1ccc(O)c(O)c1	11			
(-)-cathinone	C[C@H](N)C(=O)c1ccccc1	-11			1.30
(-)-nicotine	CN1CCC[C@H]1c1cccnc1	-6		0.85	
(-)-noradrenaline	NC[C@H](O)c1ccc(O)c(O)c1	-22			0.93
(R)-carvedilol	COc1ccccc1OCCNC[C@@H](O)COc1cccc2[nH]c3ccccc3c12	82		0.84	
(R)-deprenyl	C#CCN(C)[C@@H](C)Cc1ccccc1	16			
(R)-frovatriptan	CN[C@@H]1CCc2[nH]c3ccc(C(N)=O)cc3c2C1	-5	12.9		30.30
(R)-tolterodine	Cc1ccc(O)c([C@H](CCN(C(C)C)C(C)C)c2ccccc2)c1	50			
(S)-carvedilol	COc1ccccc1OCCNC[C@H](O)COc1cccc2[nH]c3ccccc3c12	72		0.94	
(S)-prenalterol	CC(C)NC[C@H](O)COc1ccc(O)cc1	-3	20.8		5.30
1-(4-Chlorophenyl)biguanide	NC(N)=NC(N)=Nc1ccc(Cl)cc1	12		10.80	
1380575-45-0	Cc1cc(N)c(OC(C)C)cc1C1CCNCC1	34		2.70	
1-methylxanthine	Cn1c(=O)[nH]c2nc[nH]c2c1=O	-13		0.84	
2-Methylbutyroylcarnitine	CCC(C)C(=O)OC(CC(=O)[O-])C[N+](C)(C)C	0		0.76	
2-phenylethylamine	NCCc1ccccc1	4			1.76
3-methoxy-p-tyramine	COc1cc(CCN)ccc1O	-3	32.7		3.82
4-hydroxydebrisoquine	N=C(N)N1Cc2ccccc2[C@@H](O)C1	-6		12.20	
4-methoxy-m-tyramine	COc1ccc(CCN)cc1O	-2			1.73
5,7-dihydroxytryptamine	NCCc1c[nH]c2c(O)cc(O)cc12	0			
5-Methoxy-DL-tryptophan	COc1ccc2[nH]cc(C[C@H](N)C(=O)O)c2c1	-12		1.00	
5-Methoxytryptamine	COc1ccc2[nH]cc(CCN)c2c1	12	34.6		3.75
6-alpha-methylprednisolone	CC1CC2C(C(O)CC3(C)C2CCC3(O)C(=O)CO)C2(C)C=CC(=O)C=C12	3			
6-hydroxydopamine	NCCc1cc(O)c(O)cc1O	9			
762240-09-5	c1cnn(C2CCNCC2)c1	21		2.30	
acebutolol_rac	CCCC(=O)Nc1ccc(OC[C@@H](O)CNC(C)C)c(C(C)=O)c1	7	1.1		5.20
acetaminophen	CC(=O)Nc1ccc(O)cc1	13		0.98	
acetylcholine	CC(=O)OCC[N+](C)(C)C	-11		1.10	
acetylsalicylic acid	CC(=O)Oc1ccccc1C(=O)O	-24			
acyclovir	Nc1nc2c(ncn2COCCO)c(=O)[nH]1	10		1.20	
adrenaline_rac	CNC[C@H](O)c1ccc(O)c(O)c1	7			1.00
agmatine	NCCCCN=C(N)N	-5		0.74	
alfuzosin	COc1cc2nc(N(C)CCCNC(=O)[C@@H]3CCCO3)nc(N)c2cc1OC	56		1.80	
amantadine	NC12C[C@H]3C[C@@H](C1)C[C@@H](C2)C3	10		1.10	
amifampridine	Nc1ccncc1N	-4	5.2		6.80
amiodarone	CCCCc1oc2ccccc2c1C(=O)c1cc(I)c(OCCN(CC)CC)c(I)c1	15			
amisulpride	CCN1CCC[C@@H]1CNC(=O)c1cc(S(=O)(=O)CC)c(N)cc1OC	16		7.90	1.90
amitriptyline	CN(C)CCC=C1c2ccccc2CCc2ccccc21	44			1.20

Drug	SMILES	Inhibition of ASP uptake [%]	Inhibition of sumatriptan uptake [%]	Uptake ratio [OCT1 / EV] from this publication	Uptake ratio [OCT1 / EV], prior data from our lab
amlodipine	CCOC(=O)C1=C(COCCN)NC(C)=C(C(=O)OC)[C@@H]1c1ccccc1Cl	2			
amphetamine	C[C@H](N)Cc1ccccc1	12			1.40
ampicillin	CC1(C)SC2C(NC(=O)C(N)c3ccccc3)C(=O)N2C1C(=O)O	11		1.30	
amprolium	CCCc1ncc(C[n+]2ccccc2C)c(N)n1	11			
androstanolone	CC12CCC3C(CCC4CC(=O)CCC43C)C1CCC2O	33			
atenolol_rac	CC(C)NC[C@H](O)COc1ccc(CC(N)=O)cc1	1	-9.5		5.60
atropine	CN1C2CCC1CC(OC(=O)C(CO)c1ccccc1)C2	33	69.4	3.60	
befunolol_rac	CC(=O)c1cc2cccc(OC[C@@H](O)CNC(C)C)c2o1	12		1.40	
benserazide	N[C@@H](CO)C(=O)NNCc1ccc(O)c(O)c1O	14		1.20	
benzyltriethylammonium	CC[N+](CC)(CC)Cc1ccccc1	-5	29.9		67.50
berberine	COc1ccc2cc3[n+](cc2c1OC)CCc1cc2c(cc1-3)OCO2	8	68.4	8.70	
beta-estradiol	C[C@]12CC[C@@H]3c4ccc(O)cc4CC[C@H]3[C@@H]1CC[C@@H]2O	6			
betaine	C[N+](C)(C)CC(=O)[O-]	3			1.00
betaxolol_rac	CC(C)NC[C@H](O)COc1ccc(CCOCC2CC2)cc1	5		1.30	
biperiden	OC(CCN1CCCCC1)(c1ccccc1)C1CC2C=CC1C2	44		1.30	
bisnorephedrine_rac	NC[C@H](O)c1ccccc1	-7			1.33
bisoprolol_rac	CC(C)NC[C@H](O)COc1ccc(COCCOC(C)C)cc1	-39			1.00
brucine	COc1cc2c(cc1OC)C13CCN4CC5=CCOC6CC(=O)N2C1C6C5CC43	9			
buformin	CCCCN=C(N)N=C(N)N	-11		15.40	
bumetanide	CCCCNc1cc(C(=O)O)cc(S(N)(=O)=O)c1Oc1ccccc1	6		1.10	
bupivacaine	CCCCN1CCCC[C@H]1C(=O)Nc1c(C)cccc1C	23		1.60	
bupropion_rac	C[C@H](NC(C)(C)C)C(=O)c1cccc(Cl)c1	16		1.80	
butylscopolamine	CCCC[N@@+]1(C)[C@H]2C[C@H](OC(=O)[C@H](CO)c3ccccc3)C[C@@H]1[C@H]1O[C@@H]21	9		16.20	
butyryl-L-carnitine	CCCC(=O)O[C@H](CC(=O)[O-])C[N+](C)(C)C	4		1.50	
cadaverine	NCCCCCN	0		1.00	
caffeine	Cn1c(=O)c2c(ncn2C)n(C)c1=O	-3			1.00
calcitriol	C=C1C(=CC=C2CCCC3(C)C2CCC3C(C)CCCC(C)(C)O)CC(O)CC1O	-5			
captopril	CC(CS)C(=O)N1CCCC1C(=O)O	9			
carnitine	C[N+](C)(C)C[C@@H](O)CC(=O)O	5		0.90	
cathine	C[C@H](N)[C@@H](O)c1ccccc1	0			1.50
ceritinib	Cc1cc(Nc2ncc(Cl)c(Nc3ccccc3S(=O)(=O)C(C)C)n2)c(OC(C)C)cc1C1CCNCC1	48			
cerivastatin	COCc1c(C(C)C)nc(C(C)C)c(/C=C\[C@@H](O)C[C@@H](O)CC(=O)O)c1-c1ccc(F)cc1	15			
chloramphenicol	O=C(NC(CO)C(O)c1ccc([N+](=O)[O-])cc1)C(Cl)Cl	0			

Drug	SMILES	Inhibition of ASP uptake [%]	Inhibition of sumatriptan uptake [%]	Uptake ratio [OCT1 / EV] from this publication	Uptake ratio [OCT1 / EV], prior data from our lab
chlorhexidine	NC(=NCCCCCCN=C(N)N=C(N)Nc1ccc(Cl)cc1)N=C(N)Nc1ccc(Cl)cc1	99		0.99	
chlorpheniramine_rac	CN(C)CC[C@@H](c1ccc(Cl)cc1)c1ccccn1	45		1.30	
chlorpromazine	CN(C)CCCN1c2ccccc2Sc2ccc(Cl)cc21	40		1.20	
chlorprothixen	CN(C)CCC=C1c2ccccc2Sc2ccc(Cl)cc21	48		1.30	
choline	C[N+](C)(C)CCO	-10			1.50
cimetidine	CN/C(=N\C#N)NCCSCc1nc[nH]c1C	36	72.8	3.80	
citalopram_rac	CN(C)CCC[C@]1(c2ccc(F)cc2)OCc2cc(C#N)ccc21	26		1.30	
clenbuterol_rac	CC(C)(C)NC[C@H](O)c1cc(Cl)c(N)c(Cl)c1	17			1.45
clidinium	C[N@+]12CC[C@H](CC1)[C@@H](OC(=O)C(O)(c1ccccc1)c1ccccc1)C2	16	34.2		
clomipramine	CN(C)CCCN1c2ccccc2CCc2ccc(Cl)cc21	37		1.20	
clonidine	Clc1cccc(Cl)c1NC1=NCCN1	38		1.50	
clozapine	CN1CCN(C2=Nc3cc(Cl)ccc3Nc3ccccc32)CC1	16		1.30	
cocaine	COC(=O)C1C(OC(=O)c2ccccc2)CC2CCC1N2C	20			1.30
codeine	COc1ccc2c3c1OC1C(O)C=CC4C(C2)N(C)CCC341	34		0.93	
corticosterone	CC12CCC(=O)C=C1CCC1C2C(O)CC2(C)C(C(=O)CO)CCC12	-9		0.62	
cortisone	CC12CCC(=O)C=C1CCC1C2C(=O)CC2(C)C1CCC2(O)C(=O)CO	-1			
creatine	CN(CC(=O)O)C(=N)N	0		0.99	
creatinine	CN1CC(=O)N=C1N	3		1.10	
crizotinib	C[C@H](Oc1cc(-c2cnn(C3CCNCC3)c2)cnc1N)c1c(Cl)ccc(F)c1Cl	41		1.10	
cycloguanil	CC1(C)N=C(N)N=C(N)N1c1ccc(Cl)cc1	7	42.3		24.50
cyclophosphamide	O=[P@]1(N(CCCl)CCCl)NCCCO1	11			
cytisine	O=c1cccc2n1C[C@H]1CNCC2C1	-9		1.20	
debrisoquin	N=C(N)N1CCc2ccccc2C1	28		11.10	
decynium 22	CCN1C(=Cc2ccc3ccccc3[n+]2CC)C=Cc2ccccc21.[I-]	17			
denatonium	CC[N+](CC)(CC(=O)Nc1c(C)cccc1C)Cc1ccccc1	12	58.0		21.60
deoxyepinephrine	CNCCc1ccc(O)c(O)c1	13	6.4		10.65
desipramine	CNCCCN1c2ccccc2CCc2ccccc21	33		1.20	
desvenlafaxine_rac	CN(C)C[C@H](c1ccc(O)cc1)C1(O)CCCCC1	15		3.30	
dexamethasone	CC1CC2C3CCC4=CC(=O)C=CC4(C)C3(F)C(O)CC2(C)C1(O)C(=O)CO	4			
dextromethorphan	COc1ccc2c(c1)C13CCCCC1C(C2)N(C)CC3	52		0.76	
D-glucosamine	NC1C(O)OC(CO)C(O)C1O	2			
diclofenac	O=C(O)Cc1ccccc1Nc1c(Cl)cccc1Cl	16			
diethyltryptamine	CCN(CC)CCc1c[nH]c2ccccc12	40			1.60
diltiazem_rac	COc1ccc(C2Sc3ccccc3N(CCN(C)C)C(=O)C2OC(C)=O)cc1	40		1.30	
dimethylphenylpiperazinium	C[N+]1(C)CCN(c2ccccc2)CC1	3	-0.4		38.90
dimethyltryptamine	CN(C)CCc1c[nH]c2ccccc12	27			1.50

Drug	SMILES	Inhibition of ASP uptake [%]	Inhibition of sumatriptan uptake [%]	Uptake ratio [OCT1 / EV] from this publication	Uptake ratio [OCT1 / EV], prior data from our lab
diphenhydramine	CN(C)CCOC(c1ccccc1)c1ccccc1	32			
dipyridamole	OCCN(CCO)c1nc(N2CCCCC2)c2nc(N(CCO)CCO)nc(N3CCCCC3)c2n1	16			
disopyramide	CC(C)N(CC[C@](C(N)=O)(c1ccccc1)c1ccccn1)C(C)C	49	55.9	3.70	
dobutamine_rac	C[C@@H](CCc1ccc(O)cc1)NCCc1ccc(O)c(O)c1	72	83.8		10.50
DOI	COc1cc(C[C@H](C)N)c(OC)cc1I	41			1.50
domperidon	O=c1[nH]c2ccccc2n1CCCN1CCC(n2c(=O)[nH]c3cc(Cl)ccc32)CC1	8			
dopamine	NCCc1ccc(O)c(O)c1	-13			0.85
doxazosin	COc1cc2nc(N3CCN(C(=O)[C@@H]4COc5ccccc5O4)CC3)nc(N)c2cc1OC	61		1.30	
doxepin	CN(C)CCC=C1c2ccccc2COc2ccccc21	41		1.30	
doxycycline	C[C@H]1c2cccc(O)c2C(O)=C2C(=O)[C@]3(O)C(O)=C(C(N)=O)C(=O)[C@@H](N(C)C)[C@@H]3[C@@H](O)[C@@H]21	7			
duloxetine	CNCC[C@H](Oc1cccc2ccccc12)c1cccs1	56		1.20	
edrophonium	CC[N+](C)(C)c1cccc(O)c1	-3	8.0		42.60
eletriptan	CN1CCC[C@@H]1Cc1c[nH]c2ccc(CCS(=O)(=O)c3ccccc3)cc12	42			1.35
emtricitabine	Nc1nc(=O)n(C2CSC(CO)O2)cc1F	3		2.10	
endoxifen	CCC(=C(c1ccc(O)cc1)c1ccc(OCCNC)cc1)c1ccccc1	41		1.20	
entecavir	C=C1[C@@H](n2cnc3c(=O)[nH]c(N)nc32)C[C@H](O)[C@H]1CO	1		0.87	
ephedrine	CN[C@@H](C)[C@H](O)c1ccccc1	8			1.50
escitalopram	CN(C)CCC[C@@]1(c2ccc(F)cc2)OCc2cc(C#N)ccc21	21		1.20	
esmolol_rac	COC(=O)CCc1ccc(OC[C@@H](O)CNC(C)C)cc1	-2			1.30
esomeprazole	COc1ccc2nc([S@](=O)Cc3ncc(C)c(OC)c3C)[nH]c2c1	4			
ethanolamine	NCCO	3		2.10	
etilefrine_rac	CCNC[C@](C)(O)c1cccc(O)c1	2	6.2		4.95
etomidate	CCOC(=O)c1cncn1[C@@H](C)c1ccccc1	-6			
famotidine	NC(N)=Nc1nc(CSCCC(N)=NS(N)(=O)=O)cs1	-20	12.7		34.20
fenofibrate	CC(C)OC(=O)C(C)(C)Oc1ccc(C(=O)c2ccc(Cl)cc2)cc1	-14			
fenoldopam	Oc1ccc([C@@H]2CNCCc3c2cc(O)c(O)c3Cl)cc1	-8		1.70	
fenoterol_rac	CC(Cc1ccc(O)cc1)NCC(O)c1cc(O)cc(O)c1	68	83.0		15.42
fenpiverinium	C[N+]1(CCC(C(N)=O)(c2ccccc2)c2ccccc2)CCCCC1	9	54.3		30.90
fentanyl	CCC(=O)N(c1ccccc1)C1CCN(CCc2ccccc2)CC1	16		1.50	
flunarizine	Fc1ccc(C(c2ccc(F)cc2)N2CCN(C/C=C/c3ccccc3)CC2)cc1	-35			
fluoxetine	CNCC[C@H](Oc1ccc(C(F)(F)F)cc1)c1ccccc1	37		1.10	
fluphenazine	OCCN1CCN(CCCN2c3ccccc3Sc3ccc(C(F)(F)F)cc32)CC1	25			
fluvoxamine	COCCCCC(=NOCCN)c1ccc(C(F)(F)F)cc1	24		1.40	

Drug	SMILES	Inhibition of ASP uptake [%]	Inhibition of sumatriptan uptake [%]	Uptake ratio [OCT1 / EV] from this publication	Uptake ratio [OCT1 / EV], prior data from our lab
formoterol_rac	COc1ccc(CC(C)NCC(O)c2ccc(O)c(NC=O)c2)cc1	22	31.9		2.98
furosemide	NS(=O)(=O)c1cc(C(=O)O)c(NCc2ccco2)cc1Cl	7			
gabapentin	NCC1(CC(=O)O)CCCCC1	12		1.10	
galantamine	COc1ccc2c3c1OC1CC(O)C=CC31CCN(C)C2	15		1.40	
gamma-aminobutyric acid	NCCCC(=O)O	8			
glibenclamide	COc1ccc(Cl)cc1C(=O)NCCc1ccc(S(=O)(=O)NC(=O)NC2CCCCC2)cc1	7			
glucosamine	NC1C(O)OC(CO)C(O)C1O			1.10	
glycine	NCC(=O)O	-2		1.20	
glycochenodeoxycholic acid	C[C@H](CCC(=O)NCC(=O)O)[C@H]1CC[C@H]2[C@H]3[C@H](CC[C@@]21C)[C@@]1(C)CC[C@@H](O)C[C@H]1C[C@H]3O	-17			
glycodeoxycholic acid	C[C@H](CCC(=O)NCC(=O)O)[C@H]1CC[C@H]2[C@@H]3CC[C@@H]4C[C@H](O)CC[C@]4(C)[C@H]3C[C@H](O)[C@@]21C	-9			
glycyl-L-proline	NCC(=O)N1CCC[C@H]1C(=O)O	0			
granisetron	CN1C2CCCC1CC(NC(=O)c1nn(C)c3ccccc13)C2	2		1.30	
guanfacine	NC(N)=NC(=O)Cc1c(Cl)cccc1Cl	24	74.1		4.30
halostachine	CNC[C@H](O)c1ccccc1	-3			1.55
heptylamine	CCCCCCCN	37		1.20	
hexylamine	CCCCCCN	9			
histamine	NCCc1cnc[nH]1	-4		1.10	
histidine	N[C@@H](Cc1cnc[nH]1)C(=O)O	-7		0.85	
hordenine	CN(C)CCc1ccc(O)cc1	40			2.63
hydrochlorothiazid	NS(=O)(=O)c1cc2c(cc1Cl)NCNS2(=O)=O	2			
hydrocodone	COc1ccc2c3c1OC1C(=O)CCC4C(C2)N(C)CCC314	7		0.49	
hydrocortisone	CC12CCC(=O)C=C1CCC1C2C(O)CC2(C)C1CCC2(O)C(=O)CO	5			
hydromorphone	CN1CCC23c4c5ccc(O)c4OC2C(=O)CCC3C1C5	18		1.30	
hydroxybenzylamine	NCc1ccc(O)cc1	-8			2.55
hydroxybupropion	C[C@H](NC(C)(C)CO)C(=O)c1cccc(Cl)c1	17		1.10	
ibuprofen	CC(C)Cc1ccc([C@@H](C)C(=O)O)cc1	-3			
imipramine	CN(C)CCCN1c2ccccc2CCc2ccccc21	41		1.20	
ipratropium	CC(C)[N+]1(C)C2CCC1CC(OC(=O)C(CO)c1ccccc1)C2	14	32.4		41.90
irinotecan	CCc1c2c(nc3ccc(OC(=O)N4CCC(N5CCCCC5)CC4)cc13)-c1cc3c(c(=O)n1C2)COC(=O)[C@]3(O)CC	99		1.60	
isobutyryl-L-carnitine	CC(C)C(=O)O[C@H](CC(=O)[O-])C[N+](C)(C)C	2			
isoleucine	CC[C@H](C)[C@H](N)C(=O)O	-15		0.89	
isoniazid	NNC(=O)c1ccncc1	-2		0.94	
isoproterenol_rac	CC(C)NC[C@H](O)c1ccc(O)c(O)c1	5			2.75
isovaleryl-l-carnitine	CC(C)CC(=O)O[C@H](CC(=O)[O-])C[N+](C)(C)C	-1		1.10	

Drug	SMILES	Inhibition of ASP uptake [%]	Inhibition of sumatriptan uptake [%]	Uptake ratio [OCT1 / EV] from this publication	Uptake ratio [OCT1 / EV], prior data from our lab
ketamine	CN[C@@]1(c2ccccc2Cl)CCCCC1=O	-6		1.90	1.10
ketoconazole	CC(=O)N1CCN(c2ccc(OCC3COC(Cn4ccnc4)(c4ccc(Cl)cc4Cl)O3)cc2)CC1	30		1.10	
ketoprofen	C[C@@H](C(=O)O)c1cccc(C(=O)c2ccccc2)c1	-16		0.79	
kynurenine	Nc1ccccc1C(=O)C[C@H](N)C(=O)O	6		1.20	
labetalol_rac	CC(CCc1ccccc1)NCC(O)c1ccc(O)c(C(N)=O)c1	5			1.00
l-alanine	C[C@H](N)C(=O)O	-13		0.91	
lamivudine	Nc1ccn(C2CSC(CO)O2)c(=O)n1	10		3.00	
lamotrigine	Nc1nnc(-c2cccc(Cl)c2Cl)c(N)n1	1		1.20	
lansoprazole_rac	Cc1c(OCC(F)(F)F)ccnc1C[S@](=O)c1nc2ccccc2[nH]1	-11			
L-arginine	NC(N)=NCCC[C@H](N)C(=O)O	-8		0.90	
L-asparagine	NC(=O)C[C@H](N)C(=O)O	8		0.90	
L-aspartic acid	N[C@@H](CC(=O)O)C(=O)O	9		1.20	
l-carnosine	NCCC(=O)N[C@@H](Cc1cnc[nH]1)C(=O)O	-13		0.77	
l-citrulline	NC(=O)NCCC[C@H](N)C(=O)O	7		0.67	
l-cystine	N[C@@H](CSSC[C@H](N)C(=O)O)C(=O)O	0		1.10	
levetiracetam	CC[C@@H](C(N)=O)N1CCCC1=O	-8		1.00	
levocarnitine	C[N+](C)(C)C[C@H](O)CC(=O)O	5			
levocarnitine propionate	CCC(=O)O[C@@H](CC(=O)O)C[N+](C)(C)C	0			
levodopa	N[C@@H](Cc1ccc(O)c(O)c1)C(=O)O	8		1.20	
l-glutamic acid	N[C@@H](CCC(=O)O)C(=O)O	3		0.98	
l-glutamine	NC(=O)CC[C@H](N)C(=O)O	-12		1.00	
L-hexanoylcarnitine	CCCCCC(=O)O[C@H](CC(=O)[O-])C[N+](C)(C)C	3		1.60	
lithium	[Li]	2			
l-leucine	CC(C)C[C@H](N)C(=O)O	-2		0.93	
l-lysine	NCCCC[C@H](N)C(=O)O	-7		1.00	
l-methionine	CSCC[C@H](N)C(=O)O	0		0.70	
l-methionine sulfoxide	CS(=O)CC[C@H](N)C(=O)O	-4		1.20	
l-phenylalanine	N[C@@H](Cc1ccccc1)C(=O)O	3		0.87	
L-phenylephrine	CNC[C@H](O)c1cccc(O)c1	2			
l-proline	O=C(O)[C@@H]1CCCN1	1		0.96	
l-serine	N[C@@H](CO)C(=O)O	0		1.10	
l-threonine	C[C@@H](O)[C@H](N)C(=O)O	4		0.89	
l-tryptophan	N[C@@H](Cc1c[nH]c2ccccc12)C(=O)O	1		0.74	
l-tyrosine	N[C@@H](Cc1ccc(O)cc1)C(=O)O	1		0.82	
l-valine	CC(C)[C@H](N)C(=O)O	5			1.00
maprotiline	CNCCCC12CCC(c3ccccc31)c1ccccc12	39		1.30	
MBDB	CC[C@@H](Cc1ccc2c(c1)OCO2)NC	40			1.20
MDAI	NC1Cc2cc3c(cc2C1)OCO3	17			1.40
MDEA	CCN[C@@H](C)Cc1ccc2c(c1)OCO2	44			1.30
MDMA	CN[C@@H](C)Cc1ccc2c(c1)OCO2	39			2.40
memantine	CC12CC3CC(C)(C1)CC(N)(C3)C2	33		1.20	
mepenzolate	C[N+]1(C)CCC[C@H](OC(=O)C(O)(c2ccccc2)c2ccccc2)C1	6	18.5		43.00

Drug	SMILES	Inhibition of ASP uptake [%]	Inhibition of sumatriptan uptake [%]	Uptake ratio [OCT1 / EV] from this publication	Uptake ratio [OCT1 / EV], prior data from our lab
mepivacaine	Cc1cccc(C)c1NC(=O)[C@@H]1CCCCN1C	6			
meptazinol	CC[C@]1(c2cccc(O)c2)CCCCN(C)C1	47	77.3	1.20	
mercaptopurine	S=c1nc[nH]c2nc[nH]c12	6			
meropenem	CC(O)C1C(=O)N2C(C(=O)O)=C(SC3CNC(C(=O)N(C)C)C3)C(C)C12	-2			
mesalazine	Nc1ccc(O)c(C(=O)O)c1	-3			
mescaline	COc1cc(CCN)cc(OC)c1OC	20	53.3		7.80
metaiodobenzylguanidine	NC(N)=NCc1cccc(I)c1	7	42.3		10.74
metamizol	Cc1c(N(C)CS(=O)(=O)O)c(=O)n(-c2ccccc2)n1C	5			
metanephrine	CNC[C@H](O)c1ccc(O)c(OC)c1	-2	10.8		4.84
metaproterenol_rac	CC(C)NC[C@H](O)c1cc(O)cc(O)c1	-1	-16.0		12.47
metformin	CN(C)C(=N)N=C(N)N	-12			1.70
methamphetamine	CN[C@@H](C)Cc1ccccc1	39			1.50
methyldopa	C[C@](N)(Cc1ccc(O)c(O)c1)C(=O)O	-2		1.50	
methylecgonine	COC(=O)C1C(O)CC2CCC1N2C	-6			1.10
methylnicotinamide	C[n+]1cccc(C(N)=O)c1	-9		3.60	
methylphenidate	COC(=O)C(c1ccccc1)C1CCCCN1	23		1.90	
methylscopolamine	C[N+]1(C)[C@H]2CC(OC(=O)[C@H](CO)c3ccccc3)C[C@@H]1[C@H]1O[C@@H]21	12	21.5		39.20
methyltestosterone	CC12CCC(=O)C=C1CCC1C2CCC2(C)C1CCC2(C)O	36			
metoclopramide	CCN(CC)CCNC(=O)c1cc(Cl)c(N)cc1OC	22			1.80
metoprolol_rac	COCCc1ccc(OC[C@@H](O)CNC(C)C)cc1	2			
mexiletine	Cc1cccc(C)c1OC[C@H](C)N	22		4.30	
miconazole_rac	Clc1ccc(CO[C@@H](Cn2ccnc2)c2ccc(Cl)cc2Cl)c(Cl)c1	-6		1.00	
midodrine	COc1ccc(OC)c([C@@H](O)CNC(=O)CN)c1				1.60
mildronate	C[N+](C)(C)NCCC(=O)[O-]	4		0.95	
milnacipran_rac	CCN(CC)C(=O)C1(c2ccccc2)CC1CN	39	70.0	4.20	6.30
mirabegron	Nc1nc(CC(=O)Nc2ccc(CC[NH2+]C[C@H](O)c3ccccc3)cc2)cs1	2			1.70
mitoxantrone	O=C1c2c(O)ccc(O)c2C(=O)c2c(NCCNCCO)ccc(NCCNCCO)c21	3			
monocrotaline	CC1C(=O)OC2CCN3CC=C(COC(=O)C(C)(O)C1(C)O)C23	5			
morphine	CN1CCC23c4c5ccc(O)c4OC2C(O)C=CC3C1C5	55	73.2		4.30
MPP	C[n+]1ccc(-c2ccccc2)cc1	-10			24.99
m-tyramine	NCCc1cccc(O)c1	-5	21.4		5.06
nadolol	CC(C)(C)NCC(O)COc1cccc2c1CC(O)C(O)C2	8		6.50	
nalmefene	C=C1CCC2(O)C3Cc4ccc(O)c5c4C2(CCN3CC2CC2)C1O5	37			
naproxen	COc1ccc2cc([C@@H](C)C(=O)O)ccc2c1	-36			
naratriptan	CNS(=O)(=O)CCc1ccc2[nH]cc(C3CCN(C)CC3)c2c1	3	35.0		54.40
nefazodone	CCc1nn(CCCN2CCN(c3cccc(Cl)c3)CC2)c(=O)n1CCOc1ccccc1	25			
N-ethyl-lidocaine	CC[N+](CC)(CC)CC(=O)Nc1c(C)cccc1C	1	15.1		48.40

Drug	SMILES	Inhibition of ASP uptake [%]	Inhibition of sumatriptan uptake [%]	Uptake ratio [OCT1 / EV] from this publication	Uptake ratio [OCT1 / EV], prior data from our lab
nicorandil	O=C(NCCO[N+](=O)[O-])c1cccnc1	-23			
nizatidine	CN/C(=C/[N+](=O)[O-])NCCSCc1csc(CN(C)C)n1	-11		4.70	
n-methyl-2-phenylethanamine	CNCCc1ccccc1	23			1.57
n-methyl-p-tyramine	CNCCc1ccc(O)cc1	23	54.6		5.29
n-methylserotonin	CNCCc1c[nH]c2ccc(O)cc12				31.95
n-methyltryptamine	CNCCc1c[nH]c2ccccc12	38			2.05
nn-dimethylarginine	CN(C)C(N)=NCCC[C@H](N)C(=O)O	4			
noradrenaline_rac	NC[C@H](O)c1ccc(O)c(O)c1	4			
normetanephrine_rac	COc1cc([C@@H](O)CN)ccc1O	1	7.1		3.45
norphenylephrine_rac	NC[C@H](O)c1cccc(O)c1	15	2.8		2.59
nortriptyline	CNCCC=C1c2ccccc2CCc2ccccc21	56			
o-acetyl-l-carnitine	CC(=O)O[C@H](CC(=O)[O-])C[N+](C)(C)C	3		0.75	
octopamine_rac	NC[C@H](O)c1ccc(O)cc1	4	7.1		4.13
O-desmethyl-tramadol	CN(C)CC1CCCCC1(O)c1cccc(O)c1				2.40
omeprazole_rac	COc1ccc2nc([S@@](=O)Cc3ncc(C)c(OC)c3C)[nH]c2c1	-4			
ondansetron	Cc1nccn1C[C@H]1CCc2c(c3ccccc3n2C)C1=O	28			1.00
ornithine	NCCC[C@H](N)C(=O)O	-9			
oxiconazole	Clc1ccc(CON=C(Cn2ccnc2)c2ccc(Cl)cc2Cl)c(Cl)c1	1		1.40	
oxprenolol_rac	C=CCOc1ccccc1OC[C@@H](O)CNC(C)C	35			
oxycodone	COc1ccc2c3c1OC1C(=O)CCC4(O)C(C2)N(C)CCC314	15		1.00	
oxyphenonium	CC[N+](C)(CC)CCOC(=O)[C@](O)(c1ccccc1)C1CCCCC1	25	55.0	23.30	
paliperidone_rac	Cc1nc2n(c(=O)c1CCN1CCC(c3noc4cc(F)ccc34)CC1)CCC[C@@H]2O	-3			
palonosetron	O=C1c2cccc3c2C(CCC3)CN1C1CN2CCC1CC2	26		1.30	
pantoprazole_rac	COc1ccnc(C[S@](=O)c2nc3ccc(OC(F)F)cc3[nH]2)c1OC	6		0.74	
paraxanthine	Cn1c(=O)[nH]c2ncn(C)c2c1=O	-7			1.00
paroxetine	Fc1ccc(C2CCNCC2COc2ccc3c(c2)OCO3)cc1	47			
penicillin V	CC1(C)SC2C(NC(=O)COc3ccccc3)C(=O)N2C1C(=O)O	2			
pentamidine	N=C(N)c1ccc(OCCCCCOc2ccc(C(=N)N)cc2)cc1	50		1.40	
pentobarbital	CCC[C@H](C)C1(CC)C(=O)NC(=O)NC1=O	-3			
perphenazine	OCCN1CCN(CCCN2c3ccccc3Sc3ccc(Cl)cc32)CC1	30			
pethidine	CCOC(=O)C1(c2ccccc2)CCN(C)CC1	33		1.90	
phenelzine	NNCCc1ccccc1			1.60	
phenformin	NC(N)=NC(N)=NCCc1ccccc1	10	24.4	24.00	
phentermine	CC(C)(N)Cc1ccccc1	22			1.90
phenylephrine_rac	CNC[C@H](O)c1cccc(O)c1	-7	-1.3		9.96
phenylethyl alcohol	OCCc1ccccc1	3		1.10	
pimozide	O=c1[nH]c2ccccc2n1C1CCN(CCCC(c2ccc(F)cc2)c2ccc(F)cc2)CC1	-4			
pindolol_rac	CC(C)NC[C@H](O)COc1cccc2[nH]ccc12	-1		1.60	

Drug	SMILES	Inhibition of ASP uptake [%]	Inhibition of sumatriptan uptake [%]	Uptake ratio [OCT1 / EV] from this publication	Uptake ratio [OCT1 / EV], prior data from our lab
pioglitazone	CCc1ccc(CCOc2ccc(C[C@@H]3SC(=O)NC3=O)cc2)nc1	-5			
pipamperone	NC(=O)C1(N2CCCCC2)CCN(CCCC(=O)c2ccc(F)cc2)CC1	1		1.20	
piperazine	C1CNCCN1	-6		1.10	
pirbuterol	CC(C)(C)NC[C@H](O)c1ccc(O)c(CO)n1	4	6.1		62.93
piritramide	N#CC(CCN1CCC(C(N)=O)(N2CCCCC2)CC1)(c1ccccc1)c1ccccc1	1			
PMA	COc1ccc(C[C@H](C)N)cc1	21			1.30
PMMA	CN[C@@H](C)Cc1ccc(OC)cc1	43			1.70
prazosin	COc1cc2nc(N3CCN(C(=O)c4ccco4)CC3)nc(N)c2cc1OC	25		1.20	
procainamide	CCN(CC)CCNC(=O)c1ccc(N)cc1	22			
progesterone	CC(=O)C1CCC2C3CCC4=CC(=O)CCC4(C)C3CCC12C	29			
proguanil	CC(C)N=C(N)N=C(N)Nc1ccc(Cl)cc1	10		4.90	
promethazine	C[C@@H](CN1c2ccccc2Sc2ccccc21)N(C)C	29			0.90
propafenone	CCCNC[C@H](O)COc1ccccc1C(=O)CCc1ccccc1	59		1.30	
propionylcarnitine	CCC(=O)O[C@H](CC(=O)[O-])C[N+](C)(C)C	0		0.86	
propranolol_rac	CC(C)NC[C@H](O)COc1cccc2ccccc12	36		0.90	
putrescine	NCCCCN	-5		0.64	
pyrazinamide	NC(=O)c1cnccn1	-6		0.80	
pyrimethamine	CCc1nc(N)nc(N)c1-c1ccc(Cl)cc1	40		1.30	
pyrithiamine	Cc1ncc(C[n+]2cccc(CCO)c2C)c(N)n1	5			
quinine	C=C[C@H]1CN2CC[C@H]1C[C@H]2[C@H](O)c1ccnc2ccc(OC)cc12	11		1.20	
ractopamine_rac	CC(CCc1ccc(O)cc1)NCC(O)c1ccc(O)cc1	56	82.3		7.04
ranitidine	CNC(=C[N+](=O)[O-])NCCSCc1ccc(CN(C)C)o1	-3	12.2		18.10
rasagiline	C#CCN[C@@H]1CCc2ccccc21	10			
reboxetine	CCOc1ccccc1OC(c1ccccc1)C1CNCCO1	28			
reproterol	Cn1c(=O)c2c(ncn2CCCNC[C@H](O)c2cc(O)cc(O)c2)n(C)c1=O				2.20
reserpine	COC(=O)[C@H]1[C@H]2C[C@@H]3c4[nH]c5cc(OC)ccc5c4CCN3C[C@H]2C[C@@H](OC(=O)c2cc(OC)c(OC)c(OC)c2)[C@@H]1OC	-1			
rifampicin	COC1C=COC2(C)Oc3c(C)c(O)c4c(O)c(c(C=NN5CCN(C)CC5)c(O)c4c3C2=O)NC(=O)C(C)=CC=CC(C)C(O)C(C)C(O)C(C)C(OC(C)=O)C1C	15			
ritodrine	CC(NCCc1ccc(O)cc1)C(O)c1ccc(O)cc1	67	85.1		7.60
rizatriptan	CN(C)CCc1c[nH]c2ccc(Cn3cncn3)cc12	-9	22.8		4.78
rosmarinic acid	O=C(/C=C/c1ccc(O)c(O)c1)O[C@H](Cc1ccc(O)c(O)c1)C(=O)O	-6			
S(-)-carbidopa	C[C@@](Cc1ccc(O)c(O)c1)(NN)C(=O)O	0		1.10	
S,S-ethambutol	CCC(CO)NCCNC(CC)CO	7	-1.4	21.50	20.81
salbutamol_rac	CC(C)(C)NC[C@H](O)c1ccc(O)c(CO)c1	2	-8.6		8.92

Drug	SMILES	Inhibition of ASP uptake [%]	Inhibition of sumatriptan uptake [%]	Uptake ratio [OCT1 / EV] from this publication	Uptake ratio [OCT1 / EV], prior data from our lab
salsolinol	C[C@@H]1NCCc2cc(O)c(O)cc21				50.59
sarcosine	CNCC(=O)O	1		1.10	
scopolamine	CN1[C@H]2C[C@H](OC(=O)[C@@H](CO)c3ccccc3)C[C@@H]1[C@H]1O[C@H]21	15		1.80	
sematilide	CCN(CC)CCNC(=O)c1ccc(NS(C)(=O)=O)cc1	-17	-0.2		3.50
serotonin	NCCc1c[nH]c2ccc(O)cc12	4	-10.8		11.16
sertraline	CNC1CCC(c2ccc(Cl)c(Cl)c2)c2ccccc21	21			
SN38	CCc1c2c(nc3ccc(O)cc13)-c1cc3c(c(=O)n1C2)COC(=O)[C@]3(O)CC	-9			
sorafenib	CNC(=O)c1cc(Oc2ccc(NC(=O)Nc3ccc(Cl)c(C(F)(F)F)c3)cc2)ccn1	4			
sotalol_rac	CC(C)NC[C@H](O)c1ccc(NS(C)(=O)=O)cc1	-2	-3.2		4.51
spermidine	NCCCCNCCCN	8		0.84	
spermine	NCCCNCCCCNCCCN	4			
spironolactone	CC(=O)SC1CC2=CC(=O)CCC2(C)C2CCC3(C)C(CCC34CCC(=O)O4)C12	70			
succinyl-l-carnitine	C[N+](C)(C)C[C@@H](CC(=O)[O-])OC(=O)CCC(=O)O	-1			
sufentanil	CCC(=O)N(c1ccccc1)C1(COC)CCN(CCc2cccs2)CC1	52		1.20	
sulfoglycolithocholic acid	C[C@H](CCC(=O)NCC(=O)O)[C@H]1CC[C@H]2[C@@H]3CC[C@@H]4C[C@H](OS(=O)(=O)O)CC[C@]4(C)[C@H]3CC[C@@]21C	4			
sulpiride_rac	CCN1CCC[C@@H]1CNC(=O)c1cc(S(N)(=O)=O)ccc1OC	19	12.4	7.90	
sumatriptan	CNS(=O)(=O)Cc1ccc2[nH]cc(CCN(C)C)c2c1	-7		23.90	34.75
synephrine_rac	CNC[C@H](O)c1ccc(O)cc1	-7	9.9		14.02
talinolol_rac	CC(C)(C)NC[C@H](O)COc1ccc(NC(=O)NC2CCCCC2)cc1	-13		1.60	
tamoxifen	CCC(=C(c1ccccc1)c1ccc(OCCN(C)C)cc1)c1ccccc1	-4		1.40	
tapentadol	CCC(c1cccc(O)c1)C(C)CN(C)C	36		0.94	
taurine	NCCS(=O)(=O)O	-1		1.10	
taurolithocholate	C[C@H](CCC(=O)NCCS(=O)(=O)[O-])[C@H]1CC[C@H]2[C@@H]3CC[C@@H]4C[C@H](O)CC[C@]4(C)[C@H]3CC[C@@]21C	-1			
Tcpobop	Clc1cnc(Oc2ccc(Oc3ncc(Cl)cc3Cl)cc2)c(Cl)c1	-1			
terazosin	COc1cc2nc(N3CCN(C(=O)[C@@H]4CCCO4)CC3)nc(N)c2cc1OC	13			
terbutaline_rac	CC(C)(C)NC[C@H](O)c1cc(O)cc(O)c1	-10	-15.5		6.61
tetraethylammonium	CC[N+](CC)(CC)CC	-2			
theobromine	Cn1cnc2c1c(=O)[nH]c(=O)n2C	-8			
theophylline	Cn1c(=O)c2[nH]cnc2n(C)c1=O				1.00
thiamine	Cc1ncc(C[n+]2csc(CCO)c2C)c(N)n1	-2	3.4		11.60
thiamine monophosphate	Cc1ncc(C[n+]2csc(CCOP(=O)(O)O)c2C)c(N)n1	-5		1.20	
thiamine-diphosphate	Cc1ncc(C[n+]2csc(CCO[P@@](=O)(O)OP(=O)(O)O)c2C)c(N)n1	8		0.84	

Drug	SMILES	Inhibition of ASP uptake [%]	Inhibition of sumatriptan uptake [%]	Uptake ratio [OCT1 / EV] from this publication	Uptake ratio [OCT1 / EV], prior data from our lab
tiglylcarnitine	C/C=C(\C)C(=O)O[C@@H](CC(=O)[O-])C[N+](C)(C)C	-8		0.96	
timolol	CC(C)(C)NC[C@H](O)COc1nsnc1N1CCOCC1	-3			
tolterodine	Cc1ccc(O)c([C@@H](CCN(C(C)C)C(C)C)c2ccccc2)c1				1.00
tramadol	COc1cccc([C@@]2(O)CCCC[C@@H]2CN(C)C)c1	14			0.90
tranylcypromine	NC1CC1c1ccccc1	4		1.30	1.54
trimethoprim	COc1cc(Cc2cnc(N)nc2N)cc(OC)c1OC	28		2.50	
trimethylamine-N-oxide	C[N+](C)(C)[O-]	5			
trimipramine	C[C@@H](CN(C)C)CN1c2ccccc2CCc2ccccc21	38		1.20	
tropisetron	CN1C2CCC1CC(OC(=O)c1c[nH]c3ccccc13)C2	37			2.30
tryptamine	NCCc1c[nH]c2ccccc12	17			2.19
tulobuterol_rac	CC(C)(C)NC[C@H](O)c1ccccc1Cl	44		1.50	
tyramine	NCCc1ccc(O)cc1	7			2.04
urapidil_rac	COc1ccccc1N1CCN(CCCNc2cc(=O)n(C)c(=O)n2C)CC1	5			1.10
varenicline	c1cnc2cc3c(cc2n1)C1CNCC3C1	-5			
venlafaxine_rac	COc1ccc([C@@H](CN(C)C)C2(O)CCCCC2)cc1	15		1.40	
verapamil_rac	COc1ccc(CCN(C)CCC[C@@](C#N)(c2ccc(OC)c(OC)c2)C(C)C)cc1OC	50			
xylometazoline	Cc1cc(C(C)(C)C)cc(C)c1CC1=NCCN1	55		2.00	
zalcitabine	Nc1ccn(C2CCC(CO)O2)c(=O)n1	9			
ziprasidone	O=C1Cc2cc(CCN3CCN(c4nsc5ccccc45)CC3)c(Cl)cc2N1	-8			
zolmitriptan	CN(C)CCc1c[nH]c2ccc(C[C@H]3COC(=O)N3)cc12	-2	20.7		9.64
zotepine	CN(C)CCOC1=Cc2ccccc2Sc2ccc(Cl)cc21	-1			

For some analyses in this publications, additional data from previous publications [10,13] was integrated.

Table S2: OCT1 substrates with an uptake ratio ≥ 3.

**Drug**	**Uptake ratio** [uptake OCT1 / uptake EV]	**Mean uptake ratio** [uptake OCT1 / uptake EV]	**SEM** [uptake OCT1 / uptake EV]
Phenformin	24.0	24.0	-
Sumatriptan	23.9	24.4	5.8
Oxyphenonium	23.3	24.3	1.0
(*S*,*S*)-Ethambutol	21.5	21.2	0.3
Butylscopolamine	16.2	16.2	-
Buformin	15.4	15.4	-
4-OH-Debrisoquine	12.2	12.2	-
Debrisoquin	11.1	11.1	-
1-(4-Chlorophenyl)biguanide	10.8	10.8	-
Berberine	8.7	8.7	-
Sulpiride	7.9	7.9	-
Amisulpride	7.9	4.9	3.0
Nadolol	6.5	6.5	-
Proguanil	4.9	4.9	-
Nizatidine	4.7	3.9	0.8
Mexiletine	4.3	4.3	-
Milnacipran	4.2	5.3	1.1
Cimetidine	3.8	3.9	0.1
Disopyramide	3.7	2.7	1.0
Methylnicotinamide	3.6	3.6	-
Atropine	3.6	2.7	1.0
Desvenlafaxine	3.3	3.3	-
Lamivudine	3.0	3.0	-

Mean values and standard errors of the mean were calculated between laboratories if data from other laboratories was available[10,13].

Table S3: Screened OCT1 inhibitors with an ASP^+ uptake inhibition ≥ 50% and top five "negatively inhibiting" drugs.

**Drug**	**ASP^+ Inhibition** [%]	**Mean** [% inhibition]	**Inter-laboratory SEM** [% inhibition]
Irinotecan	98.9	71.4	27.6
Chlorhexidine	98.7	99.2	0.2
(*R*)-Carvedilol	82.3	82.3	-
Dobutamine	72.2	80.1	8.1
(*S*)-Carvedilol	71.6	71.6	-
Spironolactone	69.6	83.3	6.7
Fenoterol	67.9	73.5	5.5
Ritodrine	66.8	78.7	11.7
Doxazosin	60.9	77.2	8.1
Propafenone	58.6	74.7	7.9
Ractopamine	56.2	72.6	16.6
Nortriptyline	56.0	66.9	10.9
Alfuzosin	55.7	65.6	9.6
Duloxetine	55.6	52.3	3.7
Xylometazoline	55.4	65.6	10.6
Morphine	55.2	61.5	6.5
Dextromethorphan	52.0	61.5	9.5
Sufentanil	51.6	51.6	-
Pentamidine	50.4	64.9	14.9
Verapamil	50.3	68.8	10.5
(*R*)-Tolterodine	49.8	49.8	-
	•		
Nicorandil	-22.7	-22.7	-
Acetylsalicilic acid	-24.3	-7.5	9.1
Flunarizine	-35.0	-12.9	22.2
Naproxen	-35.7	-18.8	17.2
Bisoprolol	-38.8	-21.2	17.8

Mean values and standard errors of the mean were calculated between laboratories if applicable [11,16].

Table S4: Chemical descriptors for compounds (non-)inhibiting OCT1-mediated sumatriptan uptake grouped by transport.

		**Non-inhibitors** (≤ 40% inhibition)	**Inhibitors** (> 40% inhibition)
**Good substrates** (ratio ≥ 3)	N	39	16
	MW	238 (109 – 352)	279 (151 – 349)
	$LogD_{7.4}$	-1.4 (-3.5 – 0.5)	-0.2 (-1.4 – 1.2)
	TPSA	63.8 (0 – 175.8)	60.7 (29.1 – 93.0)
	$Charge_{7.4}$	1.03 (1 – 2)	0.94 (0 – 1)
	Ring count	1.6 (0 – 5)	2.1 (1 – 5)
	H bond donors	2.4 (0 – 4)	2.2 (0 – 5)
**Poor substrates** (3 > ratio ≥ 1.5)	N	2	3
	MW	249 (153 – 344)	287 (233 – 339)
	$LogD_{7.4}$	-0.7 (-1.5 – 0.0)	0.6 (-0.4 – 1.6)
	TPSA	78.7 (66.5 – 90.8)	44.2 (23.5 – 59.2)
	$Charge_{7.4}$	1.00 (1 – 1)	1.00 (1 – 1)
	Ring count	1.5 (1 – 2)	2.3 (2 – 3)
	H bond donors	3.5 (3 – 4)	1.0 (1 – 1)
